# Proteolysis of Human Thrombin Generates Novel Host Defense Peptides

**DOI:** 10.1371/journal.ppat.1000857

**Published:** 2010-04-22

**Authors:** Praveen Papareddy, Victoria Rydengård, Mukesh Pasupuleti, Björn Walse, Matthias Mörgelin, Anna Chalupka, Martin Malmsten, Artur Schmidtchen

**Affiliations:** 1 Division of Dermatology and Venereology, Department of Clinical Sciences, Lund University, Biomedical Center, Lund, Sweden; 2 SARomics AB, Lund, Sweden; 3 Division of Infection Medicine, Department of Clinical Sciences, Lund University, Biomedical Center, Lund, Sweden; 4 Department of Microbiology, Faculty of Biochemistry, Biophysics and Biotechnology, Jagiellonian University, Kraków, Poland; 5 Department of Pharmacy, Uppsala University, Biomedical Center, Uppsala, Sweden; Tufts University School of Medicine, United States of America

## Abstract

The coagulation system is characterized by the sequential and highly localized activation of a series of serine proteases, culminating in the conversion of fibrinogen into fibrin, and formation of a fibrin clot. Here we show that C-terminal peptides of thrombin, a key enzyme in the coagulation cascade, constitute a novel class of host defense peptides, released upon proteolysis of thrombin *in vitro*, and detected in human wounds *in vivo*. Under physiological conditions, these peptides exert antimicrobial effects against Gram-positive and Gram-negative bacteria, mediated by membrane lysis, as well as immunomodulatory functions, by inhibiting macrophage responses to bacterial lipopolysaccharide. In mice, they are protective against *P. aeruginosa* sepsis, as well as lipopolysaccharide-induced shock. Moreover, the thrombin-derived peptides exhibit helical structures upon binding to lipopolysaccharide and can also permeabilize liposomes, features typical of “classical” helical antimicrobial peptides. These findings provide a novel link between the coagulation system and host-defense peptides, two fundamental biological systems activated in response to injury and microbial invasion.

## Author Summary

Wounding of the skin and other epithelial barriers represents an ever-present challenge and poses a potential threat for invasive infection and sepsis. Therefore, it is not surprising that evolutionary pressure has maintained and developed multiple host defense systems, involving initial hemostasis and fibrin formation, and the subsequent action of multiple proteins and peptides of our innate immune system. In humans, the coagulation pathways and those mediating innate immune responses to infections have so far been seen as separate entities. This view is challenged by the present study, which discloses novel host defense functions of C-terminal peptides of thrombin, a key enzyme in the coagulation cascade. The thrombin-derived peptides, which are detected in human wounds and fibrin, effectively kill microbes by membrane lysis, but also exert potent immunomodulatory and anti-endotoxic functions. Importantly, these peptides protect against *P. aeruginosa* sepsis, as well as lipopolysaccharide-induced shock in animal models. Thus, from the perspective of wounding and infection, thrombin, after fulfilling its primary function by generating a first line of defense, the fibrin clot, serves an additional role by the generation of antimicrobial and anti-endotoxic host-defense peptides.

## Introduction

The innate immune system, largely based on antimicrobial peptides, provides a first line of defense against invading microbes [1], [2], [3], [4], [5]. During recent years it has become increasingly evident that many cationic and amphipathic antimicrobial peptides, such as defensins and cathelicidins, are multifunctional, also mediating immunomodulatory roles and angiogenesis [6], [7], [8], thus motivating the recent and broader definition host defense peptides (HDP) for these members of the innate immune system. The family of HDPs has recently been shown to encompass various bioactive peptides with antimicrobial activities, including proinflammatory and chemotactic chemokines [9], neuropeptides [10], peptide hormones [11], [12], growth factors [13], the anaphylatoxin peptide C3a [14], [15], and kininogen-derived peptides [16], [17], [18].

The coagulation cascade represents a fundamental host defense system activated in response to injury and infection [19], [20]. Through a series of cascade-like proteinase activation steps, thrombin is formed, leading to fibrinogen degradation and clot formation [20]. In addition, thrombin has other physiologic functions in hemostasis; i.e., mediating clot stabilization by activation of TAFI and activation of transglutaminase (FXIII), providing anticoagulant and antifibrinolytic activities in complex with thrombomodulin, and causing platelet aggregation due to PAR cleavage [19], [20]. Moreover, thrombin elicits numerous cellular responses, including increased CAM expression and growth factor and cytokine release by endothelial cells, as well as growth stimulation of both smooth muscle and fibroblast cells [20]. These pivotal functions of thrombin in host defense, its ubiquitous occurrence in blood and in fibrin networks, the high evolutionary conservation of the enzyme, as well as presence of an amphipathic, cationic and helical C-terminus in the protein [19], made us raise the question whether thrombin could constitute a source of HDPs released at sites of wounding and infection. Our results indeed show that C-terminal peptides of thrombin constitute a previously undisclosed and significant class of HDPs, generated in humans during wounding and with therapeutic potential against infection and septic shock.

## Results

### Proteolysis of prothrombin and thrombin generates antimicrobial activity

**Figure 1:**
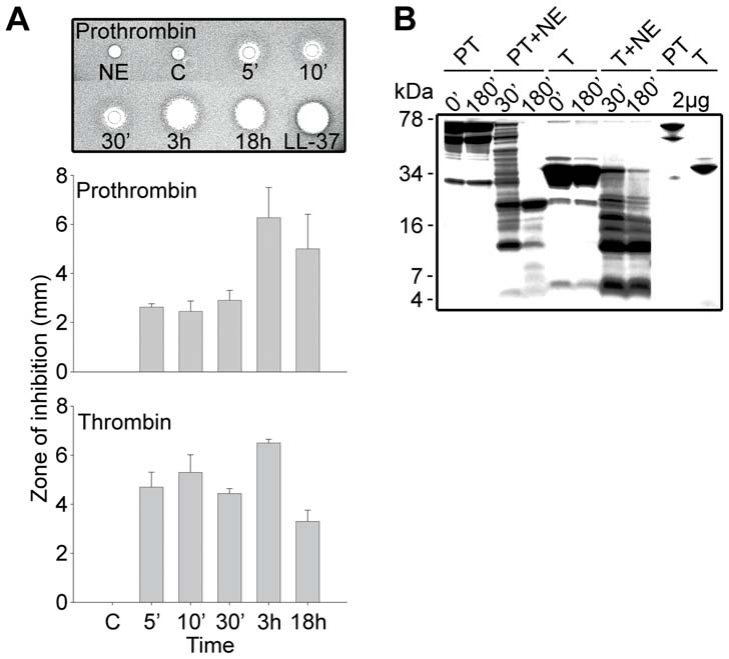
Generation of antimicrobial peptides by degradation of prothrombin and thrombin. (**A**) Degradation of the proteins was performed at 37°C for the indicated time periods. RDA was performed in low-salt conditions using *E. coli* as test organism. Each 4 mm-diameter well was loaded with 6 µl of the solution (corresponding to 3.6 µg protein). The bar diagrams indicate the diameter of the zones of clearance obtained (in mm). The inset visualizes the results obtained with prothrombin. C, buffer; NE, neutrophil elastase only. LL-37 (100 µM) was included for comparison. (**B**) Intact prothrombin (PT) and thrombin (T), and cleavage products from the different incubations with neutrophil elastase (NE, indicated above) were analyzed by SDS-PAGE (16.5% Tris-Tricine gel). The gels are overloaded (12 µg) in order to visualize generation of fragments of low molecular masses. Rightmost two lanes show PT and T proteins at 2 µg.

To test the hypothesis that prothrombin or its activated forms may generate antimicrobial peptides upon fragmentation, we incubated human prothrombin and thrombin with neutrophil elastase, a major neutral protease released by leukocytes during blood coagulation and inflammation or in response to bacterial products such as endotoxins. Earlier studies have shown that neutrophil elastase acts on proteinase-sensitive regions in human thrombin, generating smaller fragments [21]. As judged by the RDA assays (Figure 1A), digestion of the proteins yielded antimicrobial activity already after 5 min of incubation with the enzyme. In contrast, the intact mother proteins were inactive. The activity following proteolysis was still observed after several hours of incubation, suggesting the presence of relatively stable intermediates. Noteworthy, the maximum observed inhibition zones were similar in size to those generated by the classical antimicrobial peptide LL-37. Analysis by SDS-PAGE (Figure 1B) showed that the degradation generated several low molecular weight fragments in the range of 5–15 kDa. In spite of the known amidolytic properties of thrombin, no detectable antimicrobial activity was detected after prolonged incubation of the enzyme form alone (not shown). The observation that the zymogen as well as the activated forms generated similar activities, suggests that the antimicrobial epitopes localize to regions in the active enzyme after R271 (prothrombin numbering).

### Structure-based screening for identification of antimicrobial epitopes

**Figure 2:**
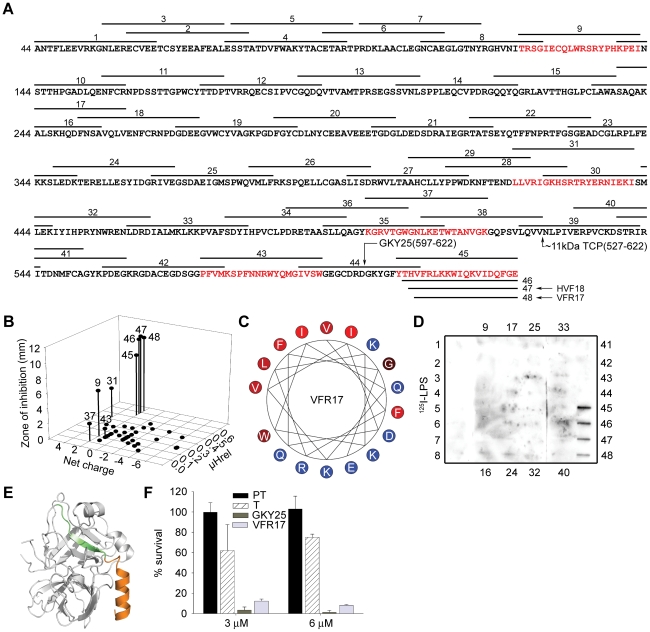
Activities of peptides derived from prothrombin. (**A**) Sequence of prothrombin and overlapping peptides (indicated by numbers). In addition to the regular overlapping peptides, peptide regions of high net charge, and/or content of predicted helical regions (Agadir; http://www.embl-heidelberg.de/Services/serrano/agadir/agadir-start.html) were selected. Peptides described in subsequent experiments are also indicated; GKY25, VFR17, and the major ∼11 kDa peptide (amino acids 527–622). (**B**) Overlapping peptides of prothrombin were analyzed for antimicrobial activities against *E. coli*. The inhibitory zones, relative hydrophobic moment (μHrel) as well as net charge of respective peptides (only active peptides are numbered) are indicated in the 3-D graph. Peptides showing antimicrobial activity are also indicated by red color in (**A**). For determination of antibacterial activities, *E. coli* (4×10^6^ cfu) was inoculated in 0.1% TSB agarose gel. Each 4 mm-diameter well was loaded with 6 µl of peptide (at 100 µM). The zones of clearance correspond to the inhibitory effect of each peptide after incubation at 37°C for 18–24 h (mean values are presented, n = 3). (**C**) Helical wheel representation of the C-terminal peptide VFR17. The amino acids are indicated. (**D**) LPS-binding activity of the prothrombin-derived peptide sequences. Peptides (5 µg) were applied to nitrocellulose membranes followed by incubation in PBS (containing 2% bovine serum albumin) with iodinated (^125^I)-LPS. Only peptides from the C-terminal part of prothrombin demonstrated significant binding to LPS. (**E**) Molecular model of thrombin. The peptides GKY25 (green-orange, indicated in Figure 2A) and VFR17 (orange, peptide 48 in Figure 2A) are indicated in the crystal structure of human thrombin (PDB code 1C5L). (**F**) Activities of prothrombin (PT), thrombin (T), GKY25 and VFR17 on *E. coli* ATCC 25922. In viable count assays GKY25 and VFR17 displayed significant antibacterial activities. 2×10^6^ cfu/ml of bacteria were incubated in 50 µl with proteins and peptides at a concentration of 3 and 6 µM, respectively.

In order to identify possible antibacterial peptide regions of prothrombin/thrombin, overlapping peptide sequences comprising 20mers (Figure 2A) were synthesized and screened for antibacterial activities against the two test bacteria *E. coli* and *P. aeruginosa*. Properties common for most antimicrobial peptides include minimum levels of cationicity, amphipathicity, and hydrophobicity [5]. Taking these structural prerequisites into account, additional peptides comprising regions of high net charge and/or presence of amphipathic helical regions, such as those encompassing the C-terminus, were selected and synthesized (Figure 2A). The experiments showed that particularly peptides derived from the C-terminal region (peptides 45–48) were antimicrobial, although other active peptides were also identified (eg. 9 and 31) (Figure 2B). However, at high ionic strength (0.1 M NaCl), only the C-terminal peptides retained their antimicrobial activity against *E. coli* as well as *P. aeruginosa* (Figure S1) demonstrating that only this region, characterized by a high relative hydrophobicity (μHrel), positive net charge (z_net_ = +2 for the most active C-terminal peptides) (Figure 2B) and amphipathicity (Figure 2C), features typical of classical antimicrobial peptides [1], [2], [3], [4], may generate peptides active against bacteria at physiologic conditions. Corresponding to the antimicrobial activities observed above, only peptides derived from the C-terminal part significantly bound to *E. coli* LPS (Figure 2D).

Since the absence of activity of the holoproteins in RDA could possibly be attributed to their high molecular weight (compromising diffusion during the assay), the antibacterial results above were further substantiated by matrix-free viable count assays. The results demonstrated that in contrast to the holoproteins, the selected model peptides VFR17 (Figure 2A; peptide 48) and the longer 25mer peptide GKY25 (indicated in Figure 2A) from the C-terminal part of the enzyme (both peptides indicated by colors in the 3D model of thrombin; Figure 2E), demonstrated significant antibacterial activity (Figure 2F), thus corroborating the RDA assays above (Figure 1A and Figure 2B). In conclusion, LPS-binding and antimicrobial data, combined with structural and biophysical considerations clearly indicate a pivotal role of Thrombin-derived C-terminal Peptides, in the following text denoted “TCP”, for mediating the antimicrobial activity.

### Definition of low molecular weight fragments generated by degradation of thrombin

**Figure 3:**
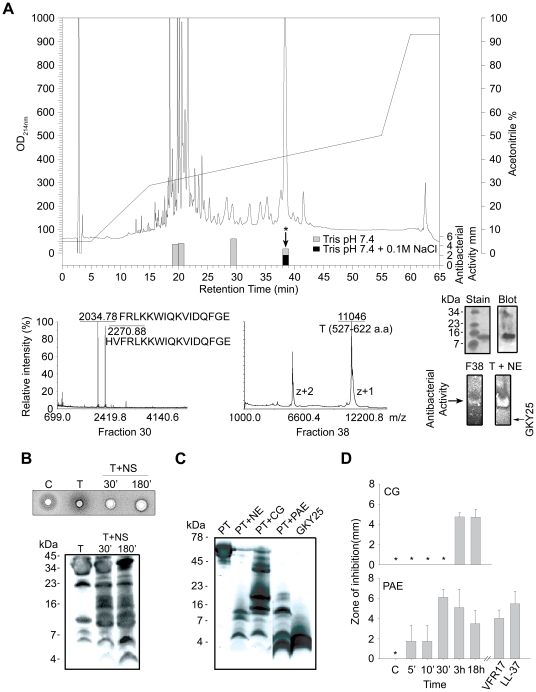
Identification of antibacterial regions of thrombin and prothrombin. (**A**) RP-HPLC separation of thrombin digested with neutrophil elastase. The bars indicate the antibacterial activity of the fractions in low (gray) as well as high salt conditions (black). Fraction 30 (lower left) contained two peaks of masses 2034.78 and 2270.88, perfectly matching the indicated sequences obtained after ESI-MS/MS analysis. Fraction 38 was analyzed by MALDI-MS, and subsequently by ESI-MS. The ESI-MS analysis identified a dominant mass of 11041 corresponding to the 96-amino acid long peptide N527-E622 (indicated in Figure 2A) with two intact disulphide bridges. A minor mass corresponding to V528-E622 was also detected by ESI-MS/MS. N- and C-terminal sequencing yelded NLPI and EGFQ, respectively. The rightmost insets illustrate the ∼11 kDa peptide analysed by SDS-PAGE and stained for protein (stain), or after immunoblot (blot), and below, the peptide (F38) was analyzed by gel-overlay for detection of antibacterial activity. The activity of F38 was identical to the major clearing zone generated by elastase-digested thrombin (T+NE). Right arrow indicates the position of clearing zone generated by the peptide GKY25. The gel was run top to bottom. Finally, peptides of fractions 20–21 were predicted using the FINDPEPT tool (www.expasy.org/tools/findpept.html) (Table S1). (**B**) Degradation of thrombin by neutrophil supernatants generates antibacterial activity in RDA (upper inset). RDA was performed in low-salt conditions. *E. coli* (4×10^6^ cfu) was used as test organism. Each 4 mm-diameter well was loaded with 6 µl of material (C, supernatant only; T, thrombin only; T+NS; thrombin incubated for 30 and 180 min respectively, with neutrophil supernatants). The digests were analysed by SDS-PAGE (16.5% Tris-Tricine gels) and immunoblotting with antibodies against VFR17 (lower panel). (**C**) Prothrombin was digested with the enzymes as indicated for 3 h, and analyzed by SDS-PAGE (16.5% Tris-Tricine gels) and immunoblotting using antibodies against VFR17 (NE, neutrophil elastase; CG, cathepsin G; PAE, *P. aeruginosa* elastase). (**D**) RDA results of prothrombin digested with cathepsin G (CG) and *P. aeruginosa* elastase (PAE) for different time periods. VFR17 and LL-37 (10 µM) are shown for comparison.

In parallel to the above structure-based screening approach, studies were undertaken to identify active fragments generated after subjection of thrombin to neutrophil elastase. RP-HPLC separation of elastase-digested thrombin, followed by antibacterial assays using *E. coli* identified several antibacterial peptide fractions (Figure 3A). Combined analyses using MALDI-TOF, ESI-MS/MS, and N- and C-terminal sequencing of fraction no. 38, which contained the majority of the activity comprising peptides active in high salt, unambiguously identified a major 11041 Da fragment comprising the C-terminal 96 amino acids of thrombin (amino acid 527–622, predicted pI 8.4, indicated in Figure 2A). Correspondingly, SDS-PAGE identified one single peptide of ∼11 kDa, that contained the C-terminal epitope, as shown by immunoblot analysis using polyclonal antibodies against the C-terminal peptide VFR17 (Figure 3A, rightmost upper panel). Gel overlay assays demonstrated that the major antimicrobial peptide of fraction 38 corresponded to one major active peptide, also identified in neutrophil elastase-digested thrombin (Figure 3A, rightmost lower panel), showing significantly lower mobility when compared to the C-terminal peptide GKY25, thus reflecting its higher molecular mass. Interestingly, MALDI-TOF and ESI-MS/MS of fraction no. 30 identified the peptide HVFRLKKWIQKVIDQFGE, described in the previous *in vitro* screening experiments (Figure 2A and B, peptide 47), as well as a shorter 16 aa long variant (Figure 3A, FRLKKWIQKVIDQFGE), both peptides from the C-terminus of thrombin. The analyses of the less hydrophobic material (fractions 20 and 21) yielded several low molecular weight fragments corresponding to internal, and cationic, sequences of low hydrophobicity and amphipathicity, matching antibacterial regions identified by the previous 20-mer screening (Table S1 available online and Figure 2A). Taken together, these results showed that neutrophil elastase generates antimicrobial TCPs, of which the major forms comprise a ∼11 kDa fragment of 96 amino acids, but also smaller fragments from the distal helical and amphipathic terminus.

### Thrombin-derived C-terminal fragments are generated by human and bacterial proteinases

During inflammation, neutrophils release a multitude of enzymes, which could have activity on either thrombin or its proform. Therefore, thrombin was incubated with supernatants from activated neutrophils and the material analysed for antimicrobial activity and generation of TCPs. Indeed, antimicrobial activity was found upon proteolysis, while immunoblotting identified several TCPs of similar molecular weights as those generated by neutrophil elastase alone (Figure 3B). Similar fragments (Figure 3C) and corresponding antimicrobial activity (Figure 3D) were also detected when prothrombin was subjected to neutrophil elastase, cathepsin G as well as the bacterial thermolysin-like proteinase of *Pseudomonas aeruginosa*, lasB (also denoted *P. aeruginosa* elastase) [22]. Interestingly, low molecular weight peptides (∼3–4 kDa), generated by the latter *P. aeruginosa* enzyme, co-migrated with the model peptide GKY25 (Figure 3C). These results demonstrated that both human and bacterial enzymes may generate TCPs, irrespective of the activation state of prothrombin.

### C-terminal thrombin peptides are generated *ex vivo* and *in vivo* and are protective against infection

**Figure 4:**
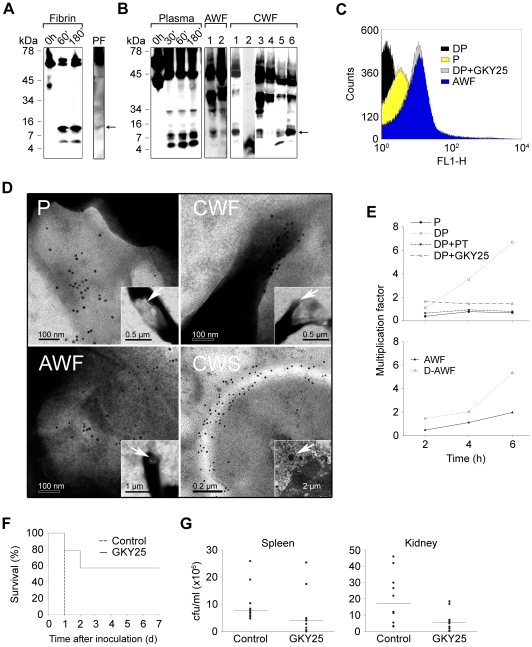
Thrombin-derived C-terminal peptides, their presence and antimicrobial effects *ex vivo* and *in vivo*. (**A**) Fibrin clots were produced from human plasma and incubated with neutrophil elastase for the indicated time periods (Fibrin), or obtained from a patient with a venous, non-infected, chronic ulcer (PF), extracted, and analyzed by immunoblotting using polyclonal antibodies against the thrombin C-terminal peptide VFR17. (**B**) Human plasma, incubated with neutrophil elastase for the indicated time periods (Plasma, left panel), acute wound fluid (patients 1–2, AWF, middle panel), or wound fluid from patients with chronic ulcers (patients 1–6, CWF, right panel) was analysed by Western blot using polyclonal antibodies against the thrombin C-terminal peptide VFR17. (**C**) Flow cytometry analysis of binding of C-terminal thrombin epitopes to *P. aeruginosa* bacteria. Bacteria were incubated for 4 h with control plasma (P), human plasma depleted of prothrombin (DP), depleted plasma supplemented with the peptide GKY25, or, acute wound fluid (AWF). Binding of C-terminal epitopes to the bacteria was detected using primary antibodies against the C-terminal epitope VFR17 followed by addition of FITC-labeled secondary antibodies. (**D**) Visualization of binding and membrane damage by TCPs. *P. aeruginosa* bacteria were incubated *ex vivo* with human plasma (P), acute wound fluid (AWF), or wound fluid from a chronic leg ulcer (CWF), or visualized *in vivo* in fibrin slough (CWS) derived from a patient with a chronic ulcer infected by *S. aureus*. Arrows in P, AWF, and CWF point to damaged bacterial membranes. Coccoid bacteria (indicated by an arrow in CWS) show extensive binding of antibodies directed against the C-terminal peptide VFR17 (negative and positive bacterial controls, and additional material are found in Figure S3 and S4). (**E**) TCPs inhibit bacterial growth in human plasma. Control plasma (P), plasma depleted of prothrombin (DP), depleted plasma supplemented with either prothrombin (DP+PT), or GKY25 (DP+GKY25) (PT and GKY25 at 1.5 µM), or control AWF or depleted AWF (D-AWF), were inoculated with *P. aeruginosa* bacteria under similar conditions as in (**C–D**). The multiplication factors at various time points are given. After incubation, CFUs were determined by plating. Experiments were repeated three times and a representative experiment is shown. (**F**) The thrombin C-terminal peptide GKY25 significantly increases survival. Mice were injected i.p. with *P. aeruginosa* bacteria, followed by subcutaneous injection of GKY25 or buffer only, after 1 h. The injections were repeated after 24 hours. Treatment with the peptide significantly increased survival (n = 10 for controls and treated, p = 0.002). (**G**) GKY25 suppresses bacterial dissemination to the spleen and kidney. Mice were infected as above, GKY25 was administrated subcutaneously after 1 h, and the cfu of *P. aeruginosa* in spleen and kidney was determined after a time period of 8 h (n = 10 for controls and treated, P<0.05 for spleen and kidney. Horizontal line indicates median value).

Prothrombin, as many other proteins in plasma, is under meticulous control by antiproteinases in the normal state, preventing its activation and/or degradation. Therefore, we hypothesized that favorable environments promoting TCP formation should comprise i) localisation as well as concentration of thrombin, and ii) local release of enzymes, such as neutrophil elastase or bacterial proteinases. These environments are typical of sites of injury and infection, such as skin wounds, comprising thrombin activation, fibrin formation, bacterial colonization or infection, and subsequent neutrophil influx. Earlier studies have shown that thrombin binds to fibrin clots, and that fibrin acts as a reservoir for active thrombin [23]. Furthermore, human neutrophils release elastase during clotting, and neutrophils also penetrate fibrin [21]. Bacteria, such as *S. aureus* and *P. aeruginosa* frequently colonize and infect skin wounds, accompanied by excessive proteolysis and activation of neutrophils [24], [25]. Given this, the production of TCPs in fibrin, as well as in sterile or infected biological fluids and wounds was investigated. The results showed that TCPs were formed when fibrin clots were subjected to neutrophil elastase *in vitro* (Figure 4A). Furthermore, a similar ∼11 kDa fragment was detected in fibrin “slough” from a patient with a non-infected chronic venous leg ulcer, indicating that TCPs can be found in fibrin *in vivo* (Figure 4A). Analogous results, showing rapid formation of TCPs, were obtained using human plasma subjected to proteolysis by neutrophil elastase, thus simulating the high elastase activity observed during wounding (Figure 4B). Importantly, TCPs were also identified in wound fluid from patients post-surgery, as well as in wound fluid from patients with chronic (non-infected) venous leg ulcers (Figure 4B). The latter wounds are always colonized by bacteria such as *S. aureus* and *P. aeruginosa* [24].

Next, a series of experiments were performed in order to study the physiological role of TCPs in relation to bacterial infection. First, initial experiments showed that immunoreactive thrombin fragments, including the ∼11 kDa TCP peptide, are proteolytically generated and exclusively bind to bacteria during *P. aeruginosa* infection of plasma and in presence of fibrin (Figure S2A). FACS analysis utilizing antibodies against the C-terminal part of thrombin showed that TCPs, either occurring in wound fluid from acute wounds, or generated in human plasma during *P. aeruginosa* infection, bind to the bacteria similarly to the above described antibacterial C-terminal peptide GKY25 (Figure 4C). Similar results were obtained using *S. aureus* (Figure S2B). Second, electron microscopy studies employing gold-labeled antibodies demonstrated that TCPs are predominantly associated with bacterial surfaces *ex vivo* and *in vivo*. Thus, *P. aeruginosa* grown in plasma or acute wound fluid exhibited disintegrated areas as shown by ejected cytoplasmic material and membrane blebs (Figure 4D, see P and AWF). Furthermore, C-terminal epitopes of thrombin were found particularly in association with these damaged zones. Similar findings were seen after incubating *P. aeruginosa* with wound fluid derived from patients with chronic ulcers (Figure 4D, CWF), as well after incubating the bacteria with the C-terminal thrombin peptide GKY25 (Figure S3). Bacteria grown in plasma or acute wound fluid supplemented with heparin (shown to block the antimicrobial effects of the two C-terminal, heparin-binding peptides of thrombin described above, GKY25 and VFR17) were similar to the control bacteria, and did not exhibit either binding of TCPs or membrane damage (Figure S3). Additionally, analysis of fibrin slough from a patient with a chronic ulcer infected with *P. aeruginosa* and *S. aureus*, identified multiple coccoid bacteria both extracellularly and inside phagocytes (Figure 4D, CWS; Figure S4), that all displayed significant binding of immunogold antibodies, demonstrating the existence of TCPs at bacterial surfaces *in vivo* in fibrin from human wounds. Figure 4E (upper panel) further shows that the growth of *P. aeruginosa* is significantly enhanced in plasma depleted of prothrombin, when compared with native control plasma. Furthermore, addition of physiological concentrations of prothrombin, or the peptide GKY25 (at 1.5 µM, equivalent to the physiological prothrombin concentration), restored the suppressive effect of plasma on bacterial growth. Acute wound fluid depleted of thrombin and C-terminal fragments also showed increased growth of *P. aeruginosa* when compared with the control (Figure 4E, lower panel). Similar results were obtained with *S. aureus* (Figure S2C). Taken together, these results, and given the above findings on the generation and binding of TCPs to bacteria *ex vivo* and *in vivo*, unequivocally demonstrate a direct link between occurrence of TCPs and suppression of bacterial growth in plasma.

Further experiments with the model C-terminal peptide GKY25, exhibiting antibacterial activities similar to the endogenously produced C-terminal peptide HVF18 (peptide 47, Figure 2A), were employed in order to further study a physiological, as well as therapeutic role of TCPs. The MIC-levels of GKY25, according to standard NCSLA-protocols, were comparable to, and in some cases lower than, those observed for LL-37 and omiganan (Table S2 available online). Since the latter is a highly active and broad-spectrum designed antimicrobial peptide now in Phase III clinical studies, the data on GKY25 also implied a possible therapeutic role for TCPs. Initial studies revealed no significant permeabilizing effects of GKY25 on human erythrocytes (60–120 µM peptide) as well as keratinocytes (up to 60 µM peptide) in plasma and serum conditions, respectively (Figure S5). In order to investigate a possible *in vivo* function of GKY25, we therefore injected this peptide subcutaneously into mice infected intraperitoneally with *P. aeruginosa*. Compared to the controls, treatment with GKY25 yielded a significant increase in survival (Figure 4F) and significantly lower bacterial numbers in the spleen and kidney of the animals (Figure 4G). Taken together, these results demonstrate that TCPs constitute a previously undisclosed neo-structure of thrombin, formed *in vitro* as well as *ex vivo* in plasma, but also *in vivo* in human wound fluid and fibrin, exerting antimicrobial activities at physiological concentrations in plasma, and finally, showing significant therapeutic potential.

### Immunomodulatory roles of TCPs

**Figure 5:**
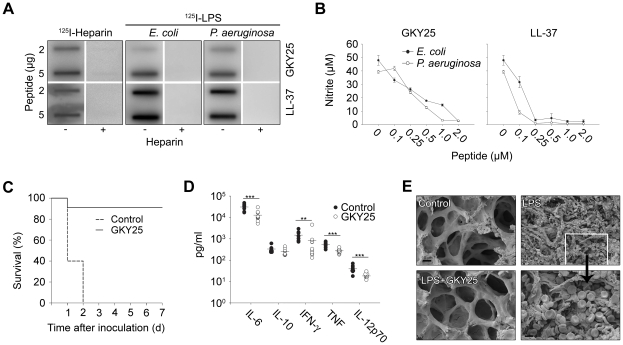
LPS-binding and immunomodulatory role *in vitro* and *in vivo* of thrombin-derived C-terminal peptides. (**A**) GKY25 binds heparin and LPS. 2 and 5 µg GKY25 were applied to nitrocellulose membranes. These membranes were then blocked in PBS (containing 2% bovine serum albumin) for 1 h at room temperature and incubated in PBS with iodinated (^125^I) heparin or LPS. Unlabeled heparin (6 mg/ml) (+) was added for competition of binding. LL-37 was used for comparison. The membranes were washed (3×10 min in PBS). A Bas 2000 radioimaging system (Fuji) was used for visualization of radioactivity. (**B**) GKY25 inhibits NO production. RAW264.7 macrophages were stimulated with LPS from *E. coli* and *P. aeruginosa*, in presence of GKY25 at the indicated concentrations. LL-37 is presented for comparison. (**C**) GKY25 significantly increases survival in LPS-induced shock. Mice were injected with LPS followed by intraperitoneal administration of GKY25 (200 µg). Survival was followed for 7 days. (n = 9 for controls, n = 10 for treated animals, P<0.001). (**D**) GKY25 attenuates proinflammatory cytokines. In a separate experiment, mice were sacrificed 6 h after i.p. injection of LPS followed by treatment with GKY25 (200 µg) or buffer, and the indicated cytokines were analyzed in blood (n = 9 for controls, n = 10 for treated animals, the P values for the respective cytokines are IL-6, 0.001; IFN-γ = 0.009; TNF, 0.001; IL-12p70, 0.001. IL-10 was not significant.). (**E**) Lungs were analyzed by scanning electron microscopy 20 h after LPS injection i.p., followed by treatment with GKY25 (200 µg) or buffer. Treatment with the peptides blocked leakage of proteins and erythrocytes (see inset) (n = 3 in both groups, and a representative lung section is shown).

As mentioned above, recent evidence shows that HDPs trigger a range of immunomodulatory responses. The observation of LPS-binding of TCPs (Figure 2D), prompted us to investigate possible endotoxin-neutralizing effects of the model peptide GKY25. Slot-binding experiments showed that the peptides bound heparin as well as LPS from *E. coli* and *P. aeruginosa* (Figure 5A). In a mouse macrophage model, GKY25 significantly inhibited NO-release of LPS-stimulated macrophages (Figure 5B), as well as release of TNF-α at concentrations below 2 µM (Figure S6). Similar effects on TNF-α were noted using human monocyte-derived macrophages (not shown). In a mouse model of LPS-induced shock, GKY25 displayed a dramatic improvement on survival (Figure 5C). Analyses of cytokines 6 hours after LPS injection, showed significant reductions of proinflammatory IL-6, IFN-γ, TNF-α, and IL-12p70, whereas IL-10 remained unchanged (Figure 5D). SEM analyses of lungs from LPS-treated animals demonstrated pulmonary leakage of protein and red blood cells (see inset in Figure 5E), an effect completely blocked by GKY25 (Figure 5E). The results thus demonstrate that GKY25, like many HDPs, is multifunctional; in addition to its antimicrobial activity it also exerts potent anti-endotoxic and immunomodulatory effects.

### Functional and structural studies of thrombin-derived C-terminal peptides

**Figure 6:**
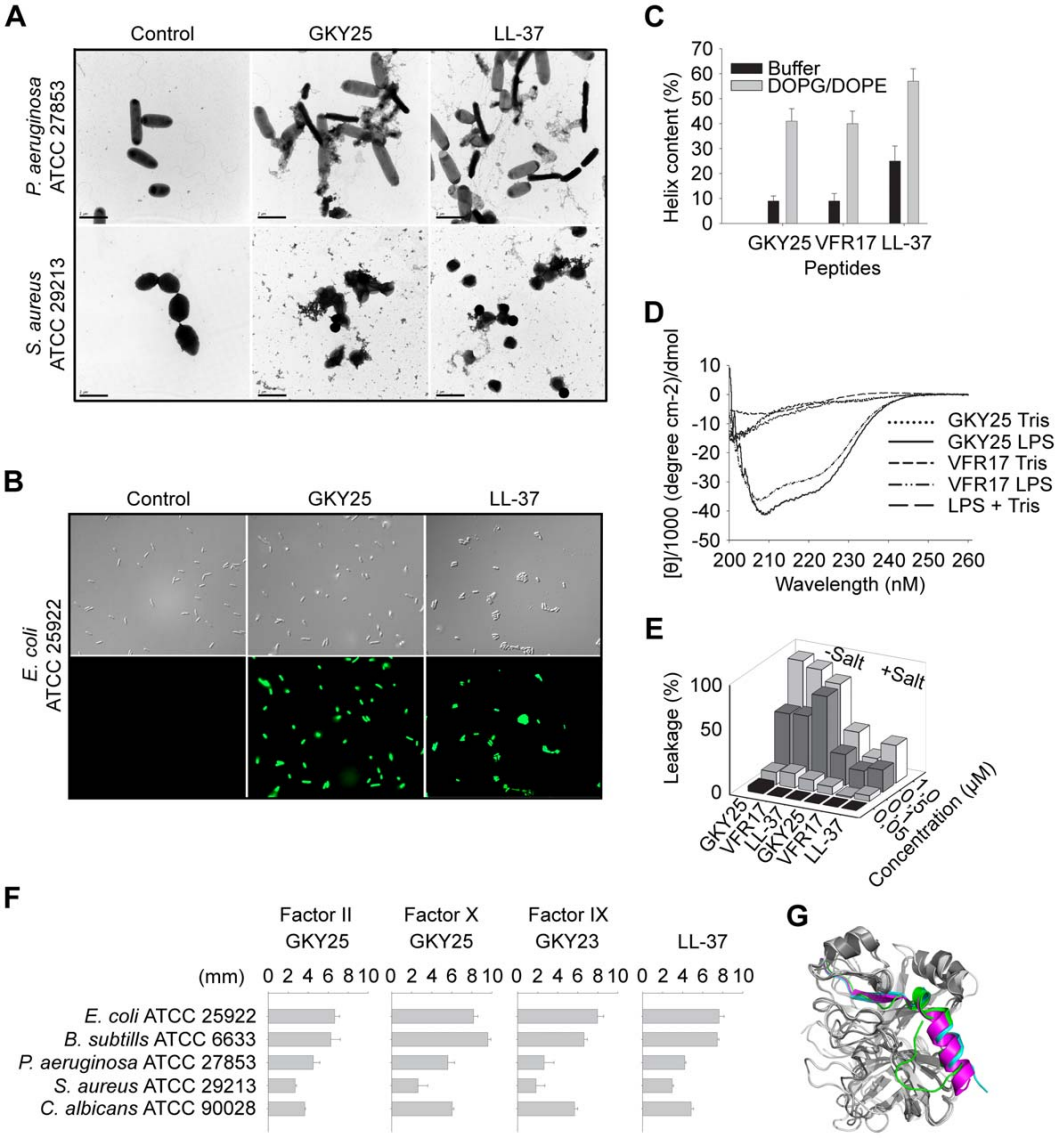
Mode of action of thrombin-derived C-terminal peptides. (**A**) Electron microscopy analysis. *P. aeruginosa* and *S. aureus* bacteria was incubated for 2 h at 37°C with 30 µM of GKY25 and LL-37 and analyzed with electron microscopy. Scale bar represents 1 µm. Control; Buffer control. (**B**) Permeabilizing effects of peptides on *E. coli*. Bacteria were incubated with the indicated peptides at 30 µM and permeabilization was assessed using the impermeant probe FITC. (**C**) Helical content of the thrombin-derived C-terminal peptides GKY25 and VFR17 in presence of negatively charged liposomes (PA). The two peptides showed a marked helix induction upon addition of the liposomes. (**D**) CD spectra of GKY25 and VFR17 in Tris-buffer and in presence of LPS. For control, CD spectra for buffer and LPS alone are also presented. (**E**) Effects of the indicated peptides on liposome leakage. The membrane permeabilizing effect was recorded by measuring fluorescence release of carboxyfluorescein from PA (negatively charged) liposomes. The experiments were performed in 10 mM Tris-buffer, in absence and presence of 0.15 M NaCl. Values represents mean of triplicate samples. (**F**) Activities of corresponding C-terminal peptides of the indicated coagulation factors. Peptides were tested in RDA against the indicated bacteria. Bacteria (4×10^6^ cfu) were inoculated in 0.1% TSB agarose gels. Each 4 mm-diameter well was loaded with 6 µl of peptide at 100 µM. The zones of clearance correspond to the inhibitory effect of each peptide after incubation at 37°C for 18–24 h. (**G**) Overlay 3D-model showing the three coagulation factors thrombin, and factor X and IX. The C-terminal parts are indicated.

To examine possible peptide-induced permeabilization of bacterial plasma membranes, *P. aeruginosa* and *S. aureus* was incubated with GKY25 at concentrations yielding complete bacterial killing (30 µM), and analyzed by electron microscopy (Figure 6A). Clear differences in morphology between peptide-treated bacteria and the control were demonstrated. The peptide caused local perturbations and breaks along *P. aeruginosa* and *S. aureus* plasma membranes, and intracellular material was found extracellularly. These findings were similar to those seen after treatment with LL-37 (Figure 6A). The data suggest that GKY25 acts on bacterial membranes, but do not demonstrate the exact mechanistic events following peptide addition to bacteria, as secondary metabolic effects on bacteria may also trigger bacterial death and membrane destabilization. However, analogous results were also obtained using the impermeant dye FITC and *E. coli* as test bacterium (Figure 6B) demonstrating membrane permeabilisation after exposure to GKY25. Kinetic studies showed that GKY25 killed >90% of bacteria after 10 minutes, compatible with a direct action on bacterial membranes (Figure S7). Furthermore, circular dichroism (CD) spectroscopy was used to study the structure and the organization of the peptides GKY25 and VFR17 in solution and on interaction with negatively charged (bacteria-like) liposomes as well as *E. coli* LPS. Neither GKY25 nor VFR17 adopted an ordered conformation in aqueous solution, however the CD spectra revealed significant structural change, largely induction of helicity, taking place in the presence of negatively charged liposomes (Figure 6C), and *E. coli* LPS (Figure 6D). Compatible with earlier results, LL-37 displayed some helicity also in buffer solution [26]. Similarly to LL-37, the two thrombin-derived peptides induced leakage of liposomes, also at high ionic strength (Figure 6E). Kinetic analysis showed that ∼80% of the maximum leakage was reached within ∼200 seconds for the two thrombin-derived peptides (at 1 µM) (not shown). Considering the above results with GKY25 and VFR17, both containing the crucial helical (and antimicrobial) epitope, the results therefore indicate that TCPs function like most helical AMPs such as LL-37 [5], [27], by interactions with both the lipid membrane and LPS (possibly also peptidoglycan) at bacterial surfaces, leading to induction of an α-helical conformation, which in turn facilitates membrane interactions, membrane destabilization, and bacterial killing.

### The TCP structure complies with a γ-core motif and is evolutionary conserved

Recently, a multidimensional signature, the γ-core motif, was identified in multiple classes of cystein-containing AMPs [28]. Analysis showed that the 96aa TCP is closely related to this fundamental motif so common in various HDPs (Figure S8). Furthermore, this region of thrombin is highly conserved in various species (Figure S9). Next, we compared the antibacterial activities of C-terminal peptide GKY25 of thrombin with corresponding peptides from other closely related human coagulation factors (Figure 6F, for sequences see Table S3 available online). Whereas the peptide from thrombin (Factor II), as well as peptides from factors X and IX were antimicrobial against Gram-negative *P. aeruginosa* and *E. coli*, Gram-positive *S. aureus* and *B. subtilis*, as well as the fungus *C. albicans* (Figure 6F), corresponding peptides from factor XI and kallikrein were inactive against these microbes (not shown). As seen in the 3D model (Figure 6G), the coagulation factors (II, X, IX) share a similar overall structure with a helical C-terminal “tail”. Indeed, C-terminals of these factors, as well as factor XI and kallikrein, contain a pattern sequence {DS}-X-[PFY]-G-[FIV]-Y-T-X-V-{C}-[AEQRY]-X-{R}-X-W-[IL]-X-{H}-X(4,24), which describes an amphipathic structure. However, only factors II, X, IX have a positive net charge (+3 or more) in this region (Table S3 available online), thus in perfect agreement with the data obtained on the antimicrobial activity (Figure 6F). Taken together, these analyses show that the TCP molecule represents a novel structural entity, which is related to other cysteine-linked HDPs containing the γ-core motif, and also found in closely related coagulation factors.

## Discussion

The key finding in this report is the discovery of a novel function of thrombin-derived C-terminal peptides in host defense. The findings expand the field of innate immunity to thrombin and the coagulation system. From an evolutionary perspective, this function of thrombin is logical, since injury and infection both represent situations necessitating an optimized innate immune system. Hence, from the perspective of wounding, thrombin, after fulfilling its primary function in generating a first line of defense, the fibrin clot, adds expanded functionality this natural physical shield by subsequent generation of antimicrobial and anti-endotoxic HDPs upon proteolysis. The significant and curative effect of a thrombin-derived peptide in a model of LPS-induced shock underscores the anti-inflammatory role this novel peptide, and contrasts to the pro-inflammatory actions of other HDPs, such as the anaphylatoxin C3a and chemotactic defensins [14], [29]. Thus, during injury and infection, different pathways are activated, employing HDPs with multiple and sometimes opposite roles, all balancing and fine-tuning inflammation while counteracting microbial invasion. Recent evidence showing a significant cross-talk between the coagulation and complement systems [30] further adds biological relevance to the observed generation of C3a and various TCPs during inflammation.

TCPs further add to the increasingly recognized redundancy of host defense mechanisms, enforcing optimized control of the microbial flora by minimizing the risk for resistance development against *one* particular HDP, as well as protecting against detrimental effects due to specific HDP deficiencies. Notably, the observation of proteolytic formation of multiple TCP fragments of different lengths parallel previous findings on LL-37 and C3a [14], [31], showing that these molecules are further processed while retaining their antimicrobial activity. Although not shown in this study, additional HDPs may be released from C-termini of factor X and XI, further increasing the arsenal of HDPs and adding to redundancy. From an investigatory standpoint, such concerted action is challenging when it comes to defining roles of a given peptide *in vivo*. Nevertheless, the suppressive effects of formed TCPs on bacterial growth *ex vivo*, their association with bacterial surfaces *ex vivo* and *in vivo*, as well as significant effects of the TCP GKY25 under standard NCSLA conditions, as well as in an animal model of *P. aeruginosa* sepsis, clearly indicate an *in vivo* role for released TCPs. These findings, in concert with the anti-endotoxic and immunomodulatory effects of the peptides *in vitro* and *in vivo*, infer interesting therapeutic possibilities for TCPs in treatment of local and systemic infections, as well as sepsis. Recent evidence, showing a higher susceptibility to *S. aureus* infection in mice rendered thrombin-deficient [32], is also compatible with the new role in host-defense of thrombin-derived C-terminal peptides revealed here. Of relevance is also the increased susceptibility to infection after inhibition of the contact system, linked to abrogated release of kininogen-derived HDPs [16], but possibly also due to a reduced capability to form TCPs and other antimicrobial molecules associated with fibrin networks. Of particular clinical relevance is also the finding that TCPs are detected in wound fluids from patients with acute surgical wounds, as well as in patients with non-healing wounds. The latter patient group is characterized by an excessive bacterial colonization e.g. by *P. aeruginosa*, extensive proteolysis and inflammation [24]. Although speculative, the noted absence of TCPs in some patients could therefore be indicative of a defective host-defense and diminished control of released endotoxins (local or systemic). Thus, apart from therapeutic possibilities, the present findings provide a potential diagnostic marker for inflammation, which is currently under evaluation in larger patient groups. Concerning the immunomodulatory role of TCPs, it should be noted that although a direct binding and thus inhibition of LPS activity was demonstrated, the observed anti-inflammatory effects could also depend on additional effects by TCPs on various intracellular signaling pathways including inhibition of NF-κB activation.

From a structural standpoint, the TCP structure relates to the previously reported γ-core signature that characterize many cysteine-containing AMPs [28], further supporting the concept of multidimensional signatures in antimicrobial peptides and extending these also to HDPs of coagulation factors. The high degree of conservation of the cysteine-constrained TCP-molecule during evolution also suggests that the TCP structure is, although novel to science, not necessarily new. Interestingly, the 96-amino acid TCP also contains a peptide region responsible for the well-known growth promoting activity of thrombin [33], further adding biological importance to the findings. It remains to be investigated whether thrombin fragments comprising this region promote cell-growth. If so, these TCPs, generated in response to injury mediate not only microbial evasion and immunomodulation, but also wound closure, three fundamental aspects of host defense.

## Materials and Methods

### Ethics statement

The use of human wound materials was approved by the Ethics Committee at Lund University (LU 708-01, LU 509-01). Written informed consent was obtained from the participants. The animal experiments were conducted according to national guidelines (Swedish Animal Welfare Act SFS 1988:534), and were approved by the Laboratory Animal Ethics Committee of Malmö/Lund.

### Peptides and proteins

Prothrombin and thrombin were from Innovative Research, USA. The coagulation factor-derived peptides (Table S3) and omiganan (ILRWPWWPWRRK-amide) were synthesized by Biopeptide Co. The purity (>95%) and molecular weight of these peptides was confirmed by mass spectral analysis (MALDI.TOF Voyager). LL-37 (LLGDFFRKSKEKIGKEFKRIVQRIKDFLRNLVPRTES) was from Innovagen AB. 20mer peptides corresponding to various overlapping regions of prothrombin (Figure 2) were from Sigma (Custom Peptide Libraries, SigmaGenosys).

### Biological materials

Wound fluids (100–600 µl) from patients with chronic venous leg ulcers were collected under a Tegaderm dressing for 2 h as previously described [25]. Fibrin slough from two chronic venous leg ulcers (chronic wound slough/surface, denoted CWS) was collected by a sterile spatula, and was immediately put into the fix solution for electron microscopy. Sterile wound fluids were obtained from surgical drainages after mastectomy. Collection was for 24 h, 24 to 48 h after surgery. Wound fluids were centrifuged, aliquoted and stored at −20°C.

### Microorganisms

*Escherichia coli* ATCC 25922, *Pseudomonas aeruginosa* ATCC 27853, *Pseudomonas aeruginosa* 15159, *Staphylococcus aureus* ATCC 29213, *Bacillus subtilis* ATCC 6633 bacterial isolates, and the fungal isolate *Candida albicans* ATCC 90028 were from the Department of Bacteriology, Lund University Hospital.

### Radial diffusion assay

Essentially as described earlier [34], [35], bacteria were grown to mid-logarithmic phase in 10 ml of full-strength (3% w/v) trypticase soy broth (TSB) (Becton-Dickinson). The microorganisms were then washed once with 10 mM Tris, pH 7.4. Subsequently, 4×10^6^ cfu were added to 15 ml of the underlay agarose gel, consisting of 0.03% (w/v) TSB, 1% (w/v) low electroendosmosis type (EEO) agarose (Sigma-Aldrich) and 0.02% (v/v) Tween 20 (Sigma-Aldrich). The underlay was poured into a Ø 144 mm petri dish. After agarose solidification, 4 mm-diameter wells were punched and 6 µl peptide solution of required concentration added to each well. Plates were incubated at 37°C for 3 h to allow peptide diffusion. The underlay gel was then covered with 15 ml of molten overlay (6% TSB and 1% Low-EEO agarose in distilled H_2_O). Antimicrobial activity of a peptide was visualized as a zone of clearing around each well after 18–24 h of incubation at 37°C.

### Viable count assays and analysis of bacterial growth

*E. coli* ATCC 25922 bacteria were grown to mid-logarithmic phase in Todd-Hewitt (TH) medium. Bacteria were washed and re-suspended in 10 mM Tris, pH 7.4 containing 5 mM glucose. *E. coli* ATCC 25922 (50 µl; 2×10^6^ cfu/ml) were incubated, at 37°C for 2 h with prothrombin, thrombin, GKY25, or VFR17 at 3 and 6 µM. For assessment of bacterial growth in plasma and effect of TCPs, overnight cultures (in TH) of *P. aeruginosa* 15159 and *S. aureus* ATCC 29213 bacteria were incubated (17 µl in 450 µl) with control plasma, acute wound fluid (AWF) or depleted AWF, plasma depleted of prothrombin (DP, Innovative Research), or DP supplemented with the peptide GKY25 (at 1.5 µM) for 0, 2, 4, and 6 h at 37°C. Serial dilutions of the incubation mixtures were plated on TH agar, followed by incubation at 37°C overnight and cfu determination. In order to deplete acute wound fluids (AWF) of thrombin and fragments containing C-terminal peptides, AWF was diluted with an equal volume of PBS, and passaged 5 times through an affinity column (0.3 ml, Thermo Scientific) having coupled IgG antibodies specific for VFR17 (Innovagen AB). For control, a column with rabbit IgG was used.

### Flow cytometry analysis

50 µl of overnight bacteria was added to 450 µl of human plasma, AWF, or DP either alone or supplemented with the peptide GKY25 (at 1.5 µM). Samples were incubated for 4 h at 37°C, centrifuged, washed with PBS, resuspended in 100 µl PBS with polyclonal antibodies against VFR17 (1∶100), and subsequently incubated for one hour at room temperature. Bacteria were pelleted and washed twice with PBS, incubated in 100 µl PBS with goat anti rabbit IgG FITC-labeled antibodies (1∶500, Sigma) for 30 min at room temperature and washed twice with PBS. Flow cytometry analysis (Becton-Dickinson, Franklin Lakes, NJ) was performed using a FACS-Calibur flow cytometry system equipped with a 15 mW argon laser turned a 488 nm. The bacterial population was selected by gating with appropriate settings of forward scatter (FSC) and sideward scatter (SSC).

### Slot-blot assay

LPS-binding ability of the peptides was examined by a slot-blot assay. Peptides (2 and 5 µg) were bound to nitrocellulose membranes (Hybond-C, GE Healthcare BioSciences), pre-soaked in PBS. Membranes were then blocked by 2 wt% BSA in PBS, pH 7.4, for 1 h at room temperature, and subsequently incubated with ^125^I-labeled LPS (40 µg/ml; 0.13×10^6^ cpm/µg) for 1 h in PBS. After incubation, the membranes were washed 3 times, 10 min each time, in PBS and visualized for radioactivity on Bas 2000 radioimaging system (Fuji). Unlabeled heparin (6 mg/ml) was added for competition of binding.

### Liposome preparation and leakage assay

Dry lipid films were prepared by dissolving either dioleoylphosphatidylethanolamine (Avanti Polar Lipids, Alabaster, AL) (70 mol%) and dioleoylphosphatidylglycerol (30 mol%) in chloroform, and then removing the solvent by evaporation under vacuum overnight. Subsequently, buffer solution containing 10 mM Tris, pH 7.4, either with or without additional 150 mM NaCl, was added together with 0.1 M carboxyfluorescein (CF) (Sigma). After hydration, the lipid mixture was subjected to eight freeze-thaw cycles consisting of freezing in liquid nitrogen and heating to 60°C. Unilamellar liposomes with a diameter of about 130 nm (as found with cryo-TEM; results not shown) were generated by multiple extrusions through polycarbonate filters (pore size 100 nm) mounted in a LipoFast miniextruder (Avestin). Untrapped carboxyfluorescein was then removed by filtration through two subsequent Sephadex G-50 columns with the relevant Tris buffer as eluent. Both extrusion and filtration was performed at 22°C. The CF release was monitored by fluorescence at 520 nm from a liposome dispersion (10 mM lipid in 10 mM Tris pH 7.4). An absolute leakage scale is obtained by disrupting the liposomes at the end of the experiment through addition of 0.8 mM Triton ×100 (Sigma), thereby causing 100% release and dequenching of CF. A SPEX-fluorolog 1650 0.22-m double spectrometer (SPEX Industries) was used for the liposome leakage assay.

### CD-spectroscopy

Circular dichroism (CD) spectra were measured by a Jasco J-810 Spectropolarimeter (Jasco, Easton, USA). The measurements were performed in triplicate at 37°C in a 10 mm quartz cuvette under stirring with a peptide concentration of 10 µM. The effect on peptide secondary structure of liposomes at a lipid concentration of 100 µM, and of *E. coli* LPS at a concentration of 0.02 wt%, was monitored in the range 200–250 nm. To account for instrumental differences between measurements, the background value (detected at 250 nm, where no peptide signal is present) was subtracted. Signals from the bulk solution were also corrected for.

### Fluorescence microscopy

For study of membrane permeabilization using the impermeant probe FITC, *E. coli* ATCC 25922 bacteria were grown to mid-logarithmic phase in TSB medium. The bacteria were washed and resuspended in either 10 mM Tris, pH 7.4, 10 mM glucose, to yield a suspension of 1×10^7^ cfu/ml. 100 µl of the bacterial suspension was incubated with 30 µM of the respective peptides at 30°C for 30 min. Microorganisms were then immobilized on poly (L-lysine)–coated glass slides by incubation for 45 min at 30°C, followed by addition onto the slides of 200 µl of FITC (6 µg/ml) in the appropriate buffers and incubated for 30 min at 30°C. The slides were washed and bacteria fixed by incubation, first on ice for 15 min, then in room temperature for 45 min in 4% paraformaldehyde. The glass slides were subsequently mounted on slides using Prolong Gold antifade reagent mounting medium (Invitrogen). For fluorescence analysis, bacteria and fungi were visualized using a Nikon Eclipse TE300 (Nikon, Melville, NY) inverted fluorescence microscope equipped with a Hamamatsu C4742-95 cooled CCD camera (Hamamatsu) and a Plan Apochromat ×100 objective (Olympus, Orangeburg, NY). Differential interference contrast (Nomarski) imaging was used for visualization of the microbes themselves.

### Electron microscopy

For transmission electron microscopy and visualization of peptide effects on bacteria, *P. aeruginosa* ATCC 27853 and *S. aureus* ATCC 29213 (1–2×10^6^ cfu/sample) were incubated for 2 h at 37°C with the peptide GKY25 at 30 µM. LL-37 (30 µM) was included as a control. Samples of *P. aeruginosa* and *S. aureus* suspensions were adsorbed onto carbon-coated copper grids for 2 min, washed briefly on two drops of water, and negatively stained on two drops of 0.75% uranyl formate. The grids were rendered hydrophilic by glow discharge at low pressure in air. For analysis of effects on biological fluids on bacterial integrity as well as detection of bound TCPs, *P. aeruginosa* 15159 bacteria, grown overnight in TH, were washed and resuspened in PBS (1×10^9^ cfu/ml). Equal volumes of bacterial suspension and chronic wound fluids were incubated together for 30 min at 37°C. For control, 2 µM of GKY25 was incubated with bacteria for 30 min at 37°C. In another experiment, *P. aeruginosa* 15159 bacteria were directly added to citrate plasma or AWF in the absence or presence of heparin (100 µg/ml), and further incubated for 4 h at 37°C. All the samples were centrifuged and washed with PBS and re-suspended in 4% paraformaldehyde and stored at 4°C, followed by gold labeling. Fibrin slough from patients with chronic venous ulcers (CWS) was fixed (1.5% PFA, 0.5% GA in 0.1 M phosphate buffer, pH 7.4) for 1 h at room temperature, followed by washing with 0.1 M phosphate buffer, pH 7.4. The fixed and washed samples were subsequently dehydrated in ethanol and further processed for Lowicryl embedding [36]. Sections were cut with a LKB ultratome and mounted on gold grids. For immunostaining, the grids were floated on top of drops of immune reagents displayed on a sheet of parafilm. Free aldehyde groups were blocked with 50 mM glycine, and the grids were then incubated with 5% (vol/vol) goat serum in incubation buffer (0.2% BSA-c in PBS, pH 7.6) for 15 minutes. This blocking procedure was followed by overnight incubation with 1 µg/ml of VFR17 polyclonal antibodies at 4°C. Controls without these primary antibodies were included. After washing the grids in a large volume (200 ml) of incubation buffer, floating on drops containing the gold conjugate reagents, 1 µg/ml EM goat antiRabbit IgG 10nm Au (BBI) in incubation buffer was performed for 2 h at 4°C. After further washes by an excess volume of incubation buffer, the sections were postfixed in 2% glutaraldehyde. Finally, sections were washed with distilled water and poststained with 2% uranyl acetate and lead citrate. All samples were examined with a Jeol JEM 1230 electron microscope operated at 80 kV accelerating voltage. Images were recorded with a Gatan Multiscan 791 charge-coupled device camera.

### Degradation of prothrombin and thrombin

Prothrombin and thrombin (Innovative Research) (27 µg, 0.6 mg/ml) was incubated at 37°C with human neutrophil elastase (NE) (0.6 µg, 20 units/mg) (Sigma) and prothrombin also with cathepsin G (0.5 µg, 2 units/mg) (BioCol GmbH) or *P. aeruginosa* elastase (PAE) (30 mU, a generous gift from Dr. H. Maeda, Kumamoto University, Japan) in a total volume of 45 µl 10 mM Tris, pH 7.4 for different time periods as indicated in the figures. Neutrophils were prepared by routine procedures (Polymorphprep) from blood obtained from healthy human donors. The cells were disrupted by freeze-thawing and addition of 0.3% Tween 20. Neutrophil extracts (corresponding to 4.8×10^7^ cells) were incubated at 37° with thrombin (27 µg, 0.6 mg/ml) for 180 min. The reaction was stopped by boiling at 95°C for 3 min. 6 µl (3.6 µg) of the material was analyzed by RDA and 20 µl (12 µg) fractions analyzed by SDS-PAGE using 16.5% precast Tris-tricine gels (Bio-rad), run under non-reducing or reducing conditions. The gels were stained with Coomassie brilliant blue and destained.

### Definition of cleavage products of thrombin

Peptide fragments of thrombin, digested by neutrophil elastase for 30 min, were separated by hplc (PerkinElmer Series 200) on a reversed phase column (Vydac 218TPC18, 250×4.6 mm, 5 µm) (Dalco chromtech AB). After injection, samples were eluted with a gradient of acetonitrile in 0.1% aqueous trifluroacetic acid at 1 ml per minute. Fractions were collected and stored at −80°C. Samples were freeze-dried, redissolved in water, and analyzed by RDA, SDS-PAGE, immunoblotting and gel-overlay assay (Figure 3). Active fractions were analyzed by combinations of MALDI-TOF MS, ESI-MS/MS, N- and C-terminal sequencing at the Karolinska Institutet Protein Analysis Center (PAC Stockholm). See also legend to Figure 3 for additional information.

### SDS-PAGE and immunoblotting

Prothrombin and thrombin, either intact or subjected to enzymes, were analyzed by SDS-PAGE on 16.5% Tris-tricine gels (Bio-Rad). Proteins and peptides were transferred to nitrocellulose membranes (Hybond-C). Membranes were blocked by 3% (w/v) skimmed milk, washed, and incubated for 1 h with rabbit polyclonal antibodies recognizing the peptide VFR17 (1∶800) (Innovagen AB) or rabbit antibodies of similar specificity (1∶1000) (Dako), washed three times for 10 min, and subsequently incubated (1 h) with HRP-conjugated secondary antibodies (1∶2000) (Dako), and then washed again three times, each time for 10 min. C-terminal thrombin peptides were visualized by an enhanced chemiluminesent substrate (LumiGLO®) developing system (Upstate cell signaling solutions). For identification of TCPs in human fibrin, normal citrate-plasma was supplemented with 10 mM Ca^2+^ in eppendorf tubes at 37°C overnight. The clots formed were washed three times with PBS and incubated with human neutrophil elastase (20 units/mg) for 0, 1, and 3 h at 37°C. Samples were centrifuged at 10000 RPM for 10 min, after which supernatants and pellets were separated. Samples were freeze-dried and then redissolved in 60% acetonitrile and 0.1% aqueous TFA. Pooled samples were freeze-dried, redissolved in water and analyzed by SDS-PAGE followed by immunoblotting as above. For identification of TCPs in human citrate plasma, 1.5 µl of citrate-plasma or patient would fluids were analysed by SDS-PAGE under reducing conditions, followed by immunoblotting as above. For identification of TCPs bound to bacteria, overnight cultures of *P. aeruginosa* 15159 bacteria were washed, resuspended, and incubated with human plasma or a preformed fibrin clot [37] for 4 h at 37°C. The bacterial cells were collected, washed wih PBS, and bound proteins were eluted with 0.1 M glycine-HCL, pH 2.0. The pH of the eluted material was raised to 7.5 with the addition of 1 M Tris. Eluted proteins were precipitated with 5% trichloroacetic acid (TCA) for 30 min on ice followed by centrifugation at 15 000g (4°C for 20 min). Precipitated material was dissolved in SDS sample buffer and subjected to Tris-Tricine SDS-PAGE under reducing conditions, followed by immunoblotting as above.

### Gel-overlay assay

Gel overlay assay was performed essentially as described previously [35]. Briefly, duplicate samples were run on non-denaturing acid urea (AU-PAGE) gels in 5% acetic acid at 100 V for 1 h 15 min. Bacteria were grown overnight in TH broth, inoculated, and grown until the OD was 0.4. The bacteria were washed and resuspended in 10 mM Tris, pH 7.4. Bacteria (4×10^6^) were added to 12 ml melted underlay agarose (10 mM Tris, pH 7.4, 0.03% TH broth, 1% agarose type 1 (Sigma-Aldrich)) and poured into a square petri dish. One AU gel was stained with Coomassie brilliant blue and one AU gel was washed three times for 4 min in 10 mM Tris, pH 7.4 and then placed on top of the agarose gel and incubated for 3 h at 37°C. The AU gel was then removed and an overlay agarose (6% TH broth, 1% agarose type 1) was poured on top of the underlay and incubated overnight at 37°C. To make the clearing zones more visible, the agarose was stained with Coomassie brilliant blue and then destained with water.

### Animal infection model

Animals were housed under standard conditions of light and temperature and had free access to standard laboratory chow and water. *P. aeruginosa* 15159 bacteria were grown overnight, harvested, washed in PBS, diluted in the same buffer to 2×10^8^ cfu/ml, and kept on ice until injection. Hundred microliter of the bacterial suspension were injected intraperitoneally (ip) into female BALB/c mice. Sixty minutes after the bacterial injection, 0.5 mg GKY25 or buffer alone was injected sc into the mice. This was repeated after 24 hours. In this *Pseudomonas* infection model, infected mice develop severe signs of sepsis within 1–2 days and usually do not recover from the infection. In order to study bacterial dissemination to target organs, the mice were infected as previously described and after a time period of 8 h, spleen and kidney were harvested, placed on ice, homogenized, and colony-forming units determined. The P-value was determined using the Mann-Whitney U-test. Data from three independent experiments were pooled.

### LPS effects on macrophages *in vitro*

3.5×10^5^ cells were seeded in 96-well tissue culture plates (Nunc, 167008) in phenol red-free DMEM (Gibco) supplemented with 10% FBS and antibiotics. Following 6 h of incubation to permit adherence, cells were stimulated with 100 or 10 ng/mL *E. coli* (0111∶B4) or *P. aeruginosa* LPS (Sigma), with and without peptide of various doses. The levels of NO in culture supernatants were determined after 24 hours from stimulation using the Griess reaction [38]. Briefly, nitrite, a stable product of NO degradation, was measured by mixing 50 µl of culture supernatants with the same volume of Griess reagent (Sigma, G4410) and reading absorbance at 550 nm after 15 min. Phenol-red free DMEM with FBS and antibiotics were used as a blank. A standard curve was prepared using 0–80 µM sodium nitrite solutions in ddH20.

### LPS model *in vivo*

C57BL/6 mice (8–10 weeks, 22+/−5g), divided into weight and sex matched groups, were injected intraperitoneally with 18 mg *E. coli* 0111∶B4 LPS (Sigma) per kg of body weight. Thirty minutes after LPS injection, 0.2 mg GKY25 or buffer alone was injected intraperitoneally into the mice. Survival and status was followed during seven days. For the cytokine assay, mice were sacrificed 6 h post LPS challenge, and blood was collected by cardiac puncture. For SEM, mice were sacrificed 20 h after LPS challenge, and lungs were removed and fixed.

### Cytokine assay

The cytokines IL-6, IL-10, MCP-1, INF-γ, TNF-α, and IL-12p70 were measured in plasma from LPS-infected mice (with or without GKY25 treatment) using the Cytometric bead array; mouse inflammation kit (Becton Dickinson AB) according to the manufacturer's instructions.

### Statistical analysis

Bar diagrams (RDA, VCA) are presented as mean and standard deviation, from at least three independent experiments. Animal data are presented as dot plots, with mean for normally distributed data, or median for data, which do not meet the criteria for normal distribution. Outliers were not excluded from the statistical analysis. Differences with P<0.05 were considered statistically significant.

### MIC, hemolysis, MTT, and LDH assay

MIC assay was carried out by a microtiter broth dilution method as previously described in the NCSLA guidelines [39]. Hemolysis, MTT, and LDH assays were performed as previously described [40] (See Text S1).

### Alignment of TCPs

See Text S1.

## Supporting Information

Text S1 Methods
(0.03 MB DOC)

Figure S1 Antimicrobial activities. Activities of peptides (RDA) of prothrombin-derived peptides against *P. aeruginosa* in absence and presence of 0.1 M NaCl, and against *E. coli* in 0.1 M NaCl. Each 4 mm-diameter well was loaded with 6 µl of the solution. The bar diagrams indicate the zones of clearance obtained (in mm).
(0.54 MB TIF)

Figure S2 TCPs are formed and bind to and inhibit microbes in plasma environment. (A) Overnight cultures of *P. aeruginosa* 15159 bacteria were washed, resuspended, and incubated with citrate plasma or a preformed fibrin clot for 4 h at 37°C. The bacterial cells were collected, washed with PBS, and bound proteins and corresponding supernatants were subjected to Tris-Tricine SDS-PAGE under reducing conditions, followed by immunoblotting with antibodies recognizing the C-terminal part of thrombin. 1 and 2, unbound and bound material in plasma; 3 and 4, unbound and bound material after incubation with fibrin. (B) Flow cytometry analysis of binding of C-terminal thrombin epitopes to *S. aureus* bacteria. Bacteria were incubated for 4 h with control plasma (P), human plasma depleted of prothrombin (DP), depleted plasma supplemented with the peptide GKY25, or, acute wound fluid (AWF). Binding of C-terminal epitopes to the bacteria was detected using primary antibodies against the C-terminal epitope VFR17 followed by addition of FITC-labeled secondary antibodies. Absence of detectable binding of FITC-labeled secondary antibodies to *S. aureus* in prothrombin-depleted plasma, excludes any significant influence of unspecific Protein A based interactions in this experimental system. Likewise, FITC-labeled antibodies alone did not show any significant unspecific binding. (C) TCPs inhibit growth of *S. aureus* in human plasma. Similarly as in Figure 4E, control plasma (P), plasma depleted of prothrombin (DP), depleted plasma supplemented with either prothrombin (DP+PT), or GKY25 (DP+GKY25) (both at 1.5 µM) were inoculated with *S. aureus* bacteria. The multiplication factors at various time points are given. After incubation, CFUs were determined by plating. Experiments were repeated three times and a representative experiment is shown.
(2.48 MB TIF)

Figure S3 TCPs bind and damage bacteria. Visualization of binding and membrane damage by TCPs. *P. aeruginosa* bacteria alone (Control) or after incubation with 1.5 µM of GKY25, were analyzed by electron microscopy following negative staining. P+Hep and AWF+Hep indicate the results obtained after addition of 100 µg/ml heparin during the incubation with human plasma and acute wound fluid, respectively. Absence of TCPs at bacterial surfaces as well as membrane damage was noted in the heparin-treated material. Examination of at least 50 different bacterial profiles demonstrated a significant difference between immunogold binding in P and AWF sections and corresponding material with heparin. Thus >80% of gold particles were associated with bacterial surfaces in P and AWF, whereas the material supplemented with heparin contained a low background of particles distributed unspecifically.
(9.78 MB TIF)

Figure S4 TCPs are found in human wounds. Visualization of binding of TCPs to cocci found in fibrin slough from a chronic wound surface (CWS-1 and -2) of two patients with *S. aureus* infected chronic leg ulcers. In the EM experiments, no significant unspecific binding of gold-conjugated IgG was observed. Scale bar; 200 nm.
(10.22 MB TIF)

Figure S5 Effects on eukaryotic cells. (A) Hemolytic effects of GKY25 in blood (EDTA-blood made 50% with PBS) were investigated. The cells were incubated with different concentrations of the peptide or LL-37. 2% Triton X-100 (Sigma-Aldrich) served as positive control. The absorbance of hemoglobin release was measured at λ 540 nm and is expressed as % of Triton X-100 induced hemolysis (note the scale of the y-axis). (B) HaCaT keratinocytes were subjected to GKY25 and LL-37 in presence of 20% human serum. Cell permeabilizing effects were measured by the LDH based TOX-7 kit. LDH release from the cells was monitored at λ 490 nm and was plotted as % of total LDH release. (C) The MTT-assay was used to measure viability of HaCaT keratinocytes in the presence of the indicated peptides. In the assay, MTT is modified into a dye, blue formazan, by enzymes associated with metabolic activity. The absorbance of the dye was measured at λ 550 nm.
(1.76 MB TIF)

Figure S6 TNF-α release is inhibited by GKY25. RAW264.7 macrophages were stimulated with LPS from *E. coli*, in presence of GKY25 at the indicated concentrations. LL-37 is presented for comparison.
(0.69 MB TIF)

Figure S7 Kinetics of GKY25 action. *E. coli* bacteria were grown to mid-logarithmic phase in Todd-Hewitt (TH) medium. They were then washed and diluted in 10 mM Tris, pH 7.4 containing 5 mM glucose. Following this, bacteria (50 ml; 2×10^6^ cfu/ml) were incubated, at 37°C for for 5, 10, 20, 40, 60 and 120 min with GKY25 at 6 µM in presence of 10 mM Tris, 0.15 M NaCl, pH 7.4. To quantify the bactericidal activity, serial dilutions of the incubation mixtures were plated on TH agar, followed by incubation at 37°C overnight and the number of colony-forming units was determined. 100% survival was defined as total survival of bacteria in the same buffer and under the same condition in the absence of peptide.
(0.22 MB TIF)

Figure S8 γ-core motif of TCP. Cartoon representation of the part corresponding to the C-terminal 96 amino acids of the crystal structure of thrombin (PDB code: 1C5L, amino acids 527–622). The region C_536_KDSTRIRITDNMFCAGYKP_555_ containing the proposed γ-core motif is indicated in red. Cysteines are indicated in yellow and glycines in orange. The motif corrsponds to the levomeric isoform 1 described by Yount and Yeaman (Yount, N.Y. & Yeaman, M.R. Multidimensional signatures in antimicrobial peptides. Proc Natl Acad Sci U S A 101, 7363–7368 (2004)); (NH2…[C]-[X13]-[CXG]-[X2]-P…COOH), and is quite similar to the γ-core motif found in kinocidins (Yeaman, M.R & Yount, N.Y. Unifying themes in host defence effector polypeptides. Nat Rev Microbiol. 5, 727–740 (2007)).
(1.35 MB TIF)

Figure S9 Alignment of human TCP with related thrombin sequences. The conserved cysteine residues, as well as two CXG motifs are indicated. Arrow indicates the N-terminus of the 96 amino acid peptide generated by neutrophil elastase.
(7.95 MB TIF)

Table S1 Peptide sequences of fraction 20–21. Masses were obtained by MALDI-MS analysis, and possible peptide sequences from the prothrombin sequence were deduced using the FINDPEPT tool (www.expasy.org/tools/findpept.html).
(0.03 MB DOC)

Table S2 Minimal inhibitory concentrations (MIC) of GKY25, LL-37 and omiganan against various bacterial isolates. The analysis was performed as described in Wiegand et al. [39] and according to NCSLA guidelines. Additional clinical isolates were obtained from the Department of Bacteriology, Lund University Hospital. *P. aeruginosa*, *E. coli* and *E. faecalis* isolates were initially derived from patients with chronic ulcers, *S. aureus* from patients with atopic dermatitis. The *S. pyogenes* strain AP1 was from the WHO Collaborating Center for References and Research on Streptococci (Prague, Czech Republic).
(0.04 MB DOC)

Table S3 Sequences of coagulation factor-derived peptides.
(0.02 MB DOC)
