# Supplementary material for: Proteolysis of Human Thrombin Generates Novel Host Defense Peptides
Source: PLoS Pathog. 2010 Apr 22;6(4):e1000857. doi: 10.1371/journal.ppat.1000857 (PMC2858699; doi:10.1371/journal.ppat.1000857)
Supplement: Text S1 — Methods (0.03 MB DOC) [file ppat.1000857.s001.doc]

**Supporting information**

**Methods**

**Minimal inhibitory concentration (MIC) determination**

MIC assay was carried out by a microtiter broth dilution method as previously described in the NCSLA guidelines (Wiegand, I., Hilpert, K. & Hancock, R.E. Agar and broth dilution methods to determine the minimal inhibitory concentration (MIC) of antimicrobial substances. *Nat Protoc* **3**, 163-175 (2008)). In brief, fresh overnight colonies were suspended to a turbidity of 0.5 units and further diluted in Mueller-Hinton broth (Becton Dickinson). For determination of MIC, peptides were dissolved in water at concentration 10 times higher than the required range by serial dilutions from a stock solution. Ten µl of each concentration was added to each corresponding well of a 96-well microtiter plate (polypropylene, Costar Corp.) and 90 µl of bacteria (1x10^5^) in MH medium added. The plate was incubated at 37 °C for 16-18 h. MIC was taken as the lowest concentration where no visual growth of bacteria was detected.

**Hemolysis assay**

EDTA-blood was diluted (1:1) with PBS. The cells were then incubated with end-over-end rotation for 1 h at 37°C in the presence of peptides (60 and 120 µM). 2% Triton X-100 (Sigma-Aldrich) served as positive control. The samples were then centrifuged at 800 g for 10 min. The absorbance of hemoglobin release was measured at  540 nm and is in the plot expressed as % of TritonX-100 induced hemolysis.

**Lactate dehydrogenase (LDH) assay**

HaCaT keratinocytes were grown in 96 well plates (3000 cells/well) in serum free keratinocyte medium (SFM) supplemented with bovine pituitary extract and recombinant EGF (BPE-rEGF) (Invitrogen, USA) to confluency. The medium was then removed, and 100 µl of the peptides investigated (at 3, 6, 30 and 60 µM, diluted in SFM/BPE-rEGF with 20% human serum), were added in triplicates to different wells of the plate, and incubations were performed for 16 h. The LDH based TOX-7 kit (Sigma-Aldrich, St Louis, USA) was used for quantification of LDH release from the cells. Results given represent mean values from triplicate measurements. Results are given as fractional LDH release compared to the positive control consisting of 1% Triton X-100 (yielding 100% LDH release).

**MTT assay**

Sterile filtered MTT (3-(4,5-dimethylthiazolyl)-2,5-diphenyl-tetrazolium bromide; Sigma-Aldrich) solution (5 mg/ml in PBS) was stored protected from light at -20°C until usage. HaCaT keratinocytes, 3000 cells/well, were seeded in 96 well plates and grown in keratinocyte-SFM/BPE-rEGF medium to confluency. Peptides were then added at the concentrations indicated in the figure (in the same medium supplemented with 20% human serum). After incubation for 16 h, 20 µl of the MTT solution was added to each well and the plates incubated for 1 h in CO_2_ at 37°C. The MTT containing medium was then removed by aspiration. The blue formazan product generated was dissolved by the addition of 100 µl of 100% DMSO per well. The plates were then gently swirled for 10 min at room temperature to dissolve the precipitate. The absorbance was monitored at 550 nm, and results given represent mean values from triplicate measurements.

**Alignment of TCPs**

The prothrombin amino acid sequence was retrieved from the NCBI site. Each sequence was analyzed with Psi-Blast (NCBI) to find the ortholog and paralog sequences. Sequences that showed structural homology >70% were selected. These sequences were aligned using ClustalW using Blosum 69 protein weight matrix settings. Internal adjustments were made taking the structural alignment into account utilizing the ClustalW interface. The level of consistency of each position within the alignment was estimated by using the alignment-evaluating software Tcoffee.
