# Supplementary figures and images for: Proteolysis of Human Thrombin Generates Novel Host Defense Peptides
Source: PLoS Pathog. 2010 Apr 22;6(4):e1000857. doi: 10.1371/journal.ppat.1000857 (PMC2858699; doi:10.1371/journal.ppat.1000857)

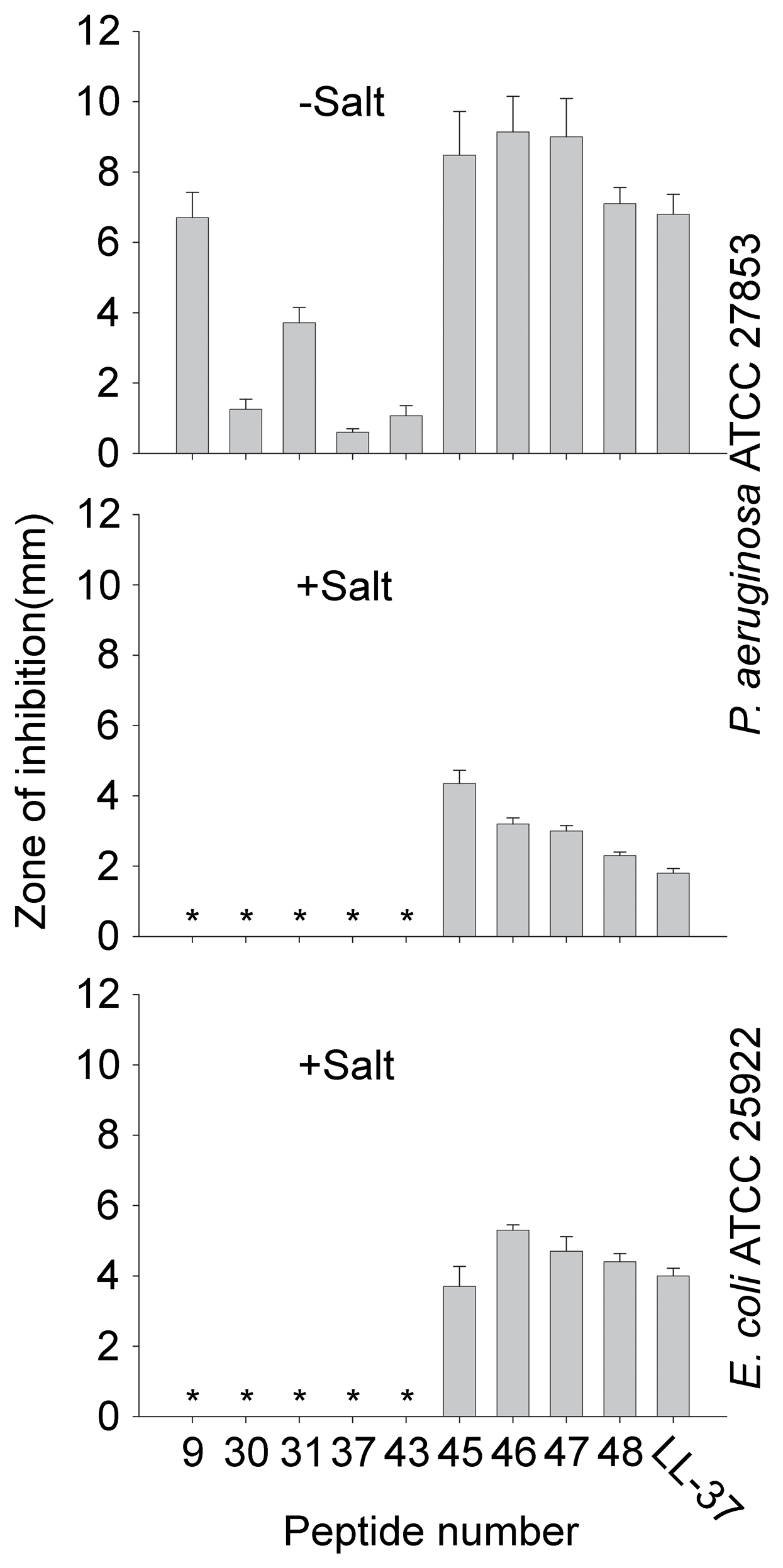

Supplement: Figure S1 — Antimicrobial activities. Activities of peptides (RDA) of prothrombin-derived peptides against P. aeruginosa in absence and presence of 0.1 M NaCl, and against E. coli in 0.1 M NaCl. Each 4 mm-diameter well was loaded with 6 µl of the solution. The bar diagrams indicate the zones of clearance obtained (in mm). (0.54 MB TIF) [file ppat.1000857.s002.tif]

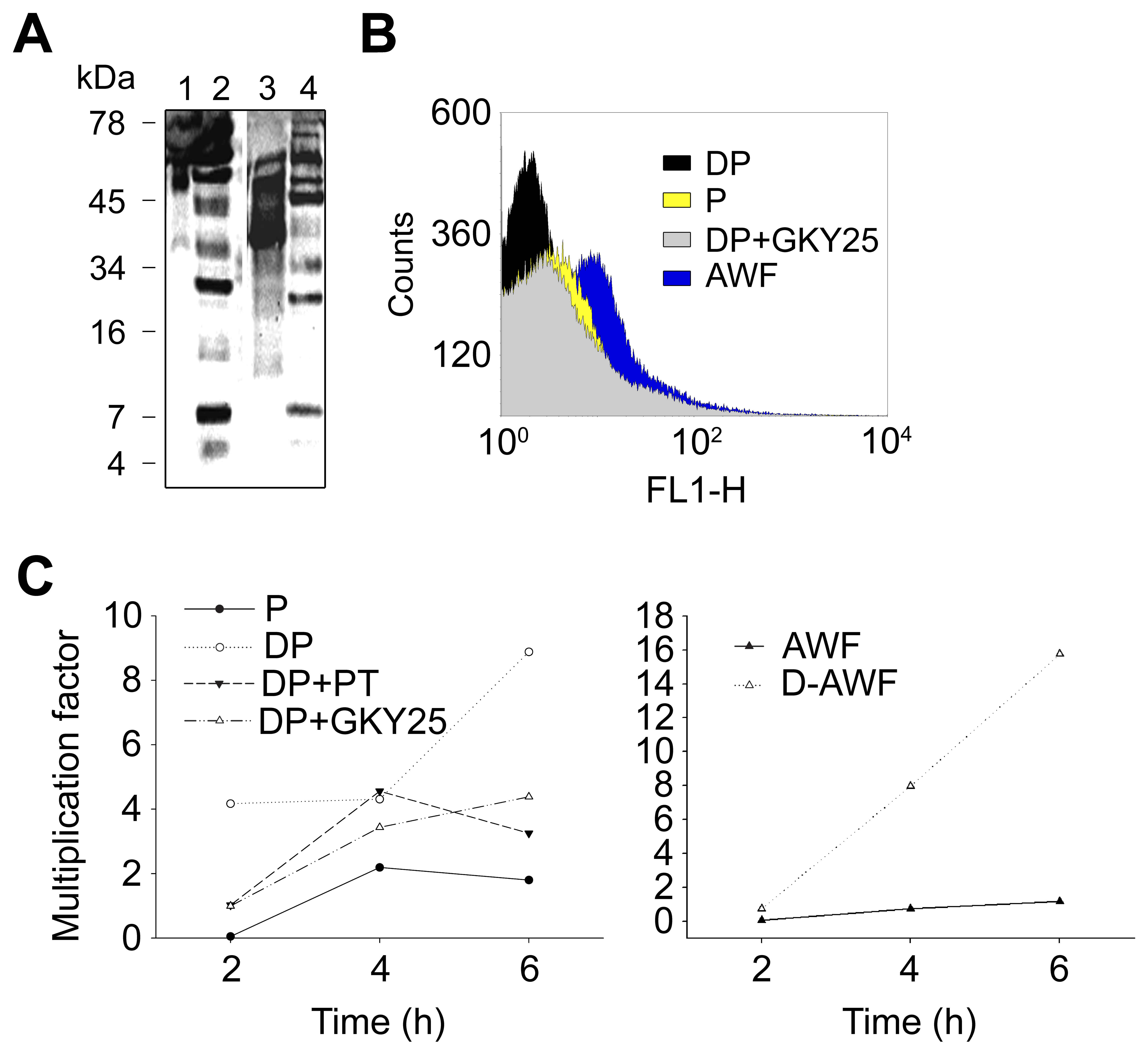

Supplement: Figure S2 — TCPs are formed and bind to and inhibit microbes in plasma environment. (A) Overnight cultures of P. aeruginosa 15159 bacteria were washed, resuspended, and incubated with citrate plasma or a preformed fibrin clot for 4 h at 37°C. The bacterial cells were collected, washed with PBS, and bound proteins and corresponding supernatants were subjected to Tris-Tricine SDS-PAGE under reducing conditions, followed by immunoblotting with antibodies recognizing the C-terminal part of thrombin. 1 and 2, unbound and bound material in plasma; 3 and 4, unbound and bound material after incubation with fibrin. (B) Flow cytometry analysis of binding of C-terminal thrombin epitopes to S. aureus bacteria. Bacteria were incubated for 4 h with control plasma (P), human plasma depleted of prothrombin (DP), depleted plasma supplemented with the peptide GKY25, or, acute wound fluid (AWF). Binding of C-terminal epitopes to the bacteria was detected using primary antibodies against the C-terminal epitope VFR17 followed by addition of FITC-labeled secondary antibodies. Absence of detectable binding of FITC-labeled secondary antibodies to S. aureus in prothrombin-depleted plasma, excludes any significant influence of unspecific Protein A based interactions in this experimental system. Likewise, FITC-labeled antibodies alone did not show any significant unspecific binding. (C) TCPs inhibit growth of S. aureus in human plasma. Similarly as in Figure 4E, control plasma (P), plasma depleted of prothrombin (DP), depleted plasma supplemented with either prothrombin (DP+PT), or GKY25 (DP+GKY25) (both at 1.5 µM) were inoculated with S. aureus bacteria. The multiplication factors at various time points are given. After incubation, CFUs were determined by plating. Experiments were repeated three times and a representative experiment is shown. (2.48 MB TIF) [file ppat.1000857.s003.tif]

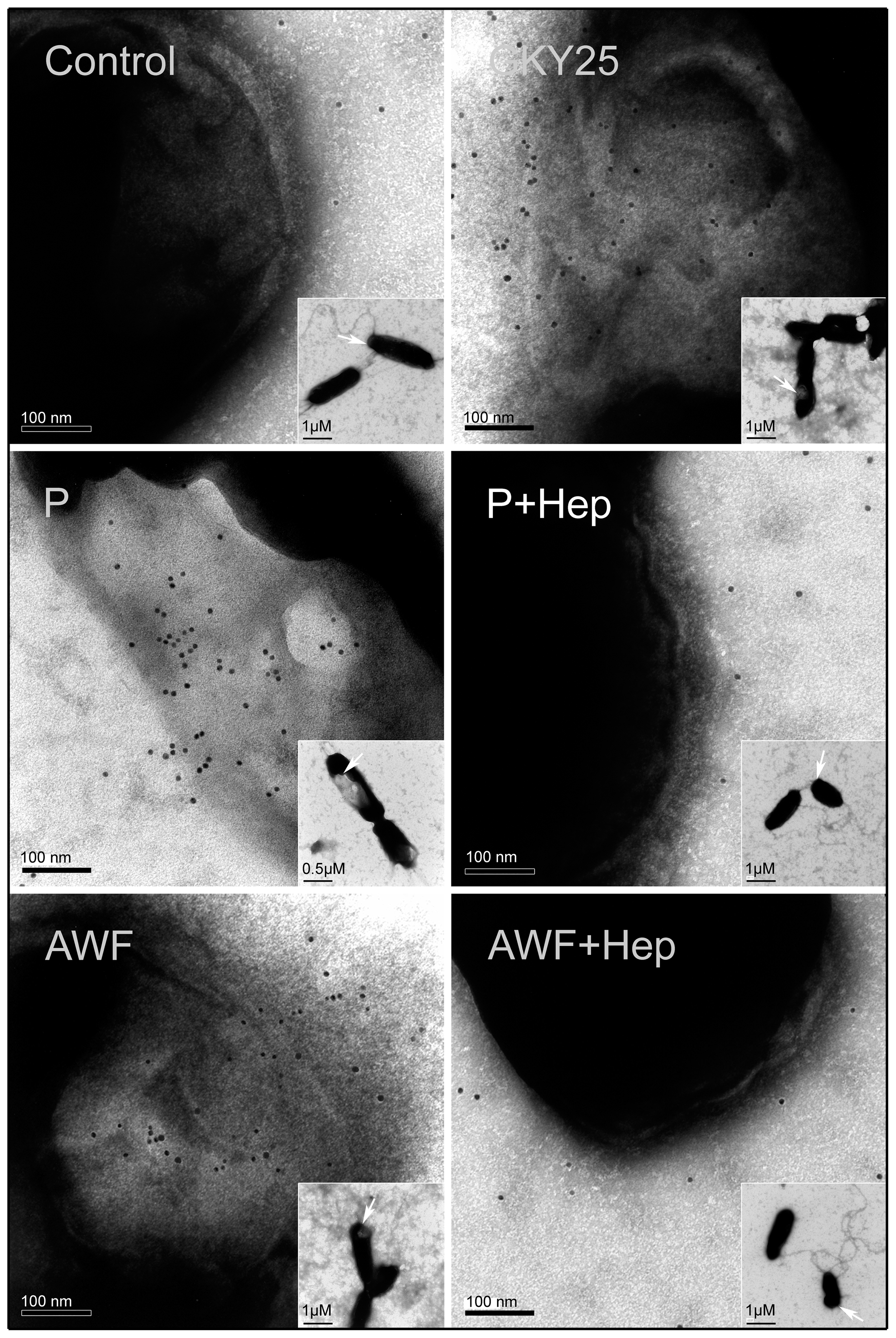

Supplement: Figure S3 — TCPs bind and damage bacteria. Visualization of binding and membrane damage by TCPs. P. aeruginosa bacteria alone (Control) or after incubation with 1.5 µM of GKY25, were analyzed by electron microscopy following negative staining. P+Hep and AWF+Hep indicate the results obtained after addition of 100 µg/ml heparin during the incubation with human plasma and acute wound fluid, respectively. Absence of TCPs at bacterial surfaces as well as membrane damage was noted in the heparin-treated material. Examination of at least 50 different bacterial profiles demonstrated a significant difference between immunogold binding in P and AWF sections and corresponding material with heparin. Thus >80% of gold particles were associated with bacterial surfaces in P and AWF, whereas the material supplemented with heparin contained a low background of particles distributed unspecifically. (9.78 MB TIF) [file ppat.1000857.s004.tif]

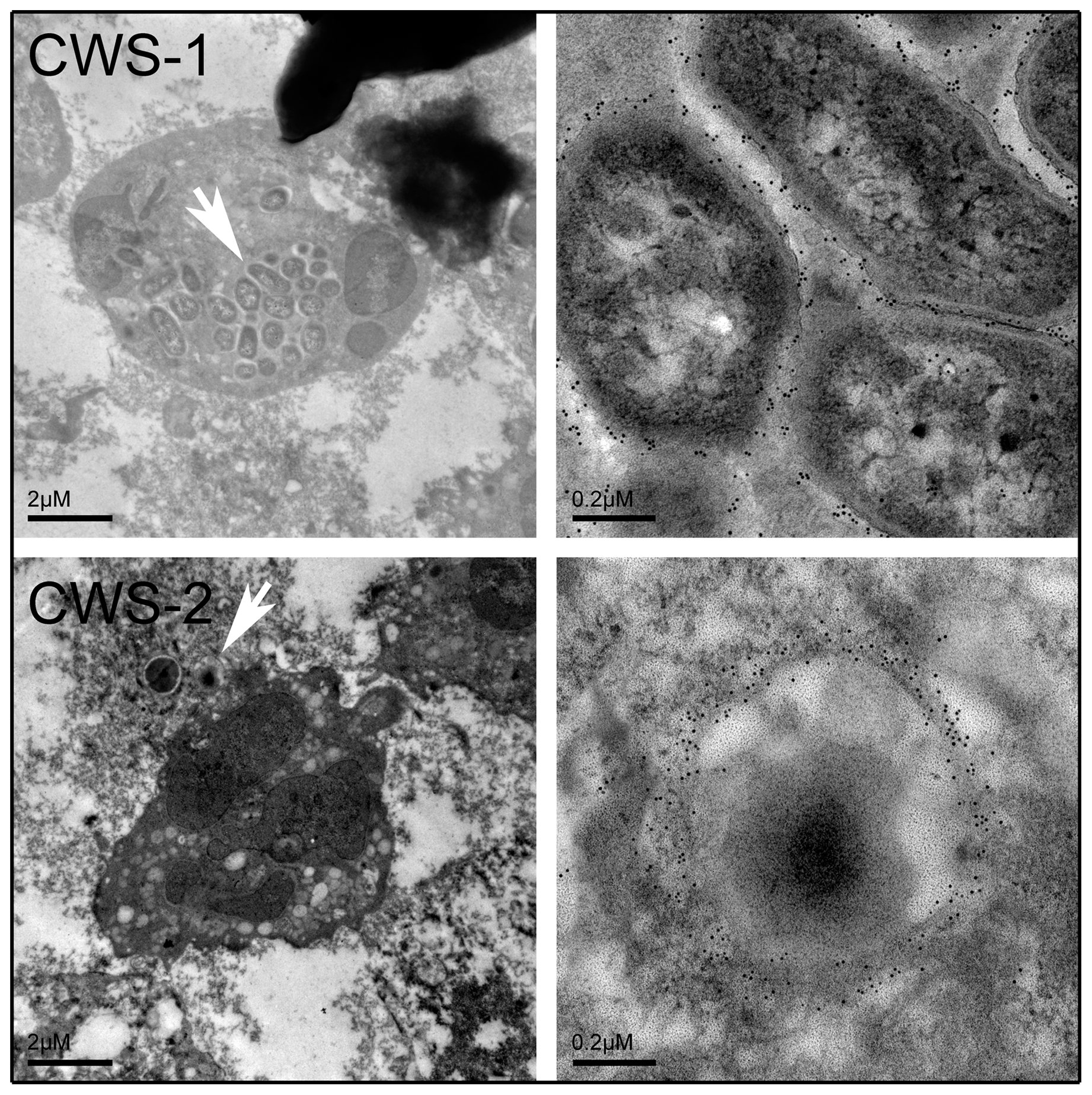

Supplement: Figure S4 — TCPs are found in human wounds. Visualization of binding of TCPs to cocci found in fibrin slough from a chronic wound surface (CWS-1 and -2) of two patients with S. aureus infected chronic leg ulcers. In the EM experiments, no significant unspecific binding of gold-conjugated IgG was observed. Scale bar; 200 nm. (10.22 MB TIF) [file ppat.1000857.s005.tif]

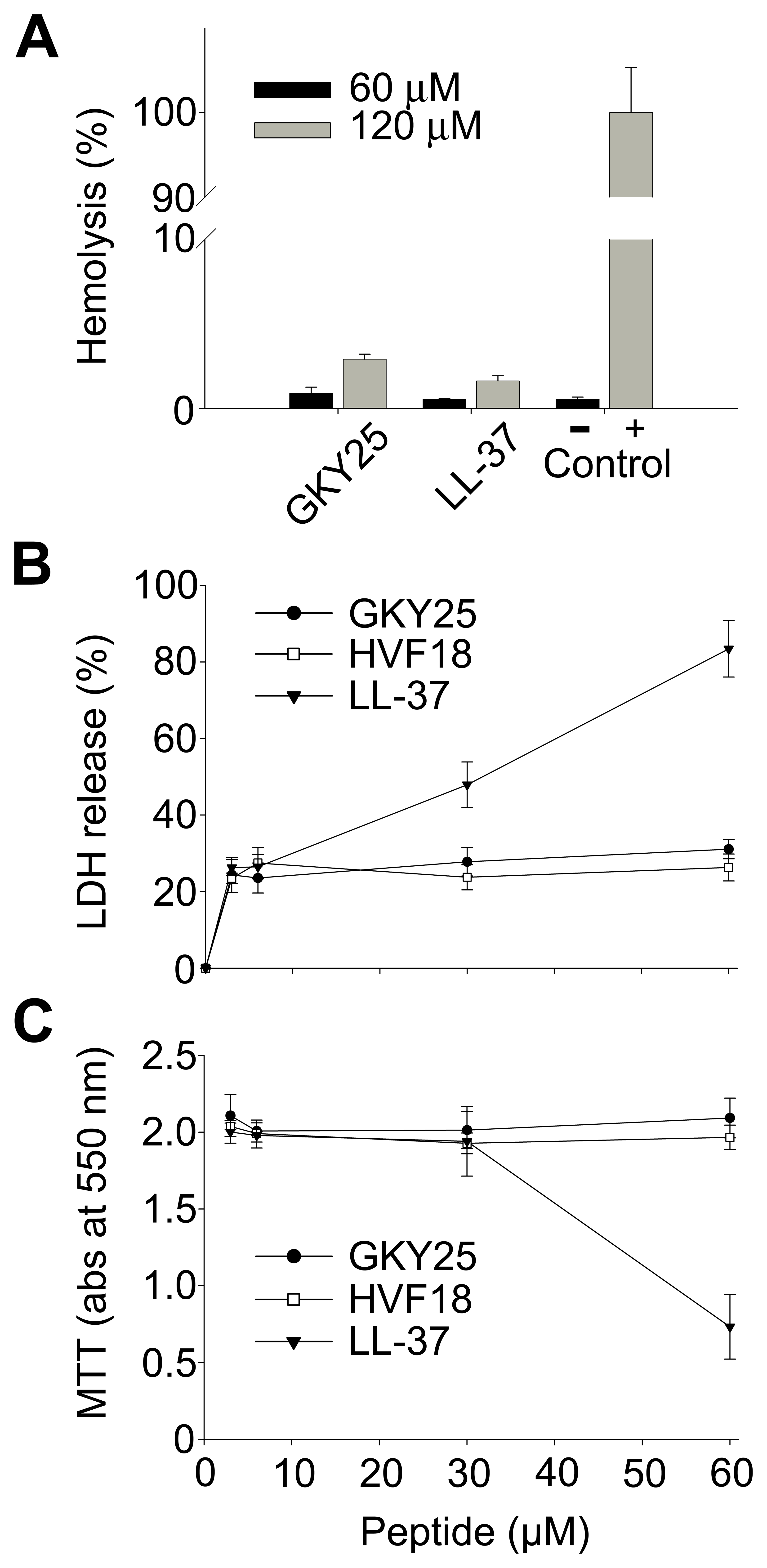

Supplement: Figure S5 — Effects on eukaryotic cells. (A) Hemolytic effects of GKY25 in blood (EDTA-blood made 50% with PBS) were investigated. The cells were incubated with different concentrations of the peptide or LL-37. 2% Triton X-100 (Sigma-Aldrich) served as positive control. The absorbance of hemoglobin release was measured at λ 540 nm and is expressed as % of Triton X-100 induced hemolysis (note the scale of the y-axis). (B) HaCaT keratinocytes were subjected to GKY25 and LL-37 in presence of 20% human serum. Cell permeabilizing effects were measured by the LDH based TOX-7 kit. LDH release from the cells was monitored at λ 490 nm and was plotted as % of total LDH release. (C) The MTT-assay was used to measure viability of HaCaT keratinocytes in the presence of the indicated peptides. In the assay, MTT is modified into a dye, blue formazan, by enzymes associated with metabolic activity. The absorbance of the dye was measured at λ 550 nm. (1.76 MB TIF) [file ppat.1000857.s006.tif]

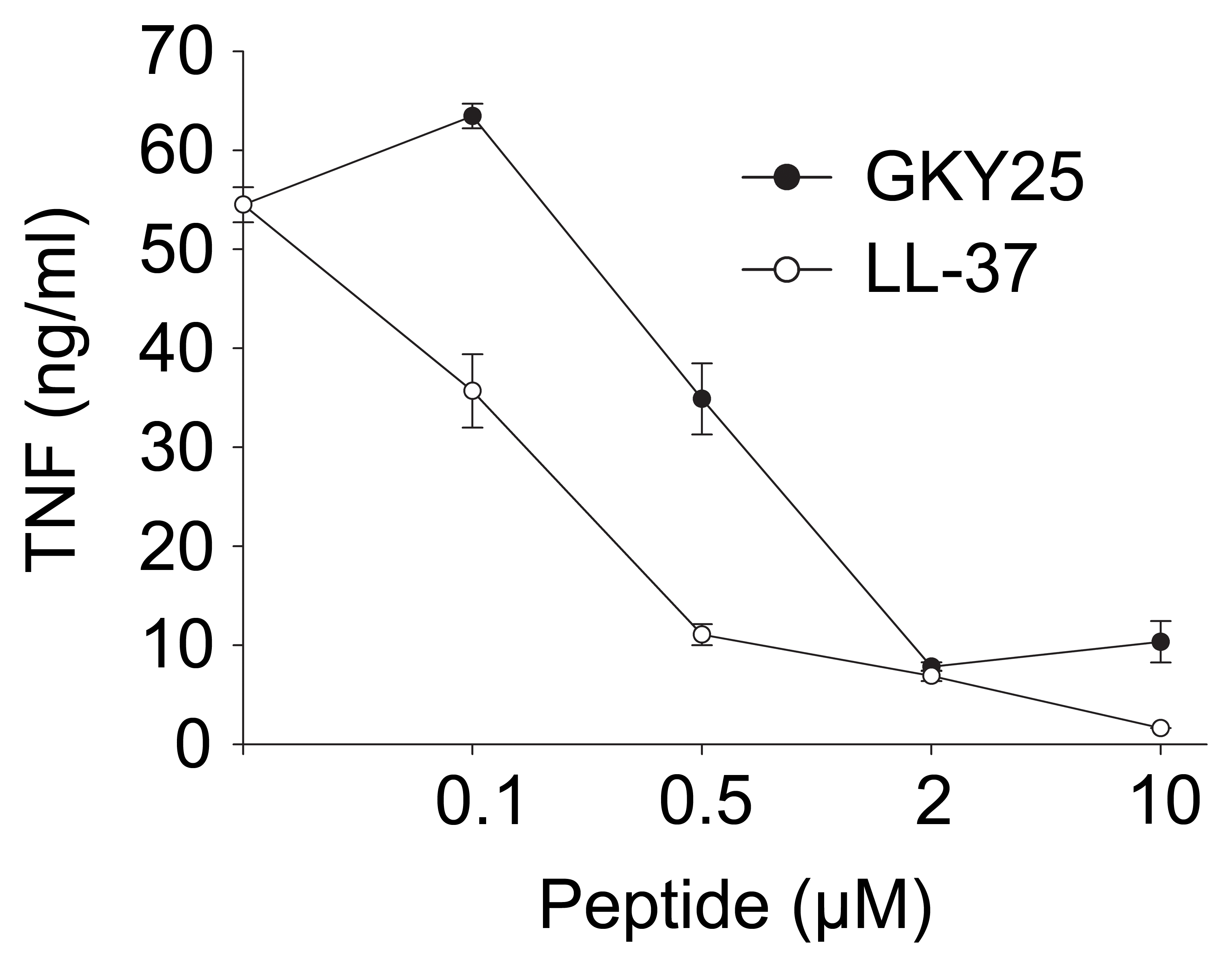

Supplement: Figure S6 — TNF-α release is inhibited by GKY25. RAW264.7 macrophages were stimulated with LPS from E. coli, in presence of GKY25 at the indicated concentrations. LL-37 is presented for comparison. (0.69 MB TIF) [file ppat.1000857.s007.tif]

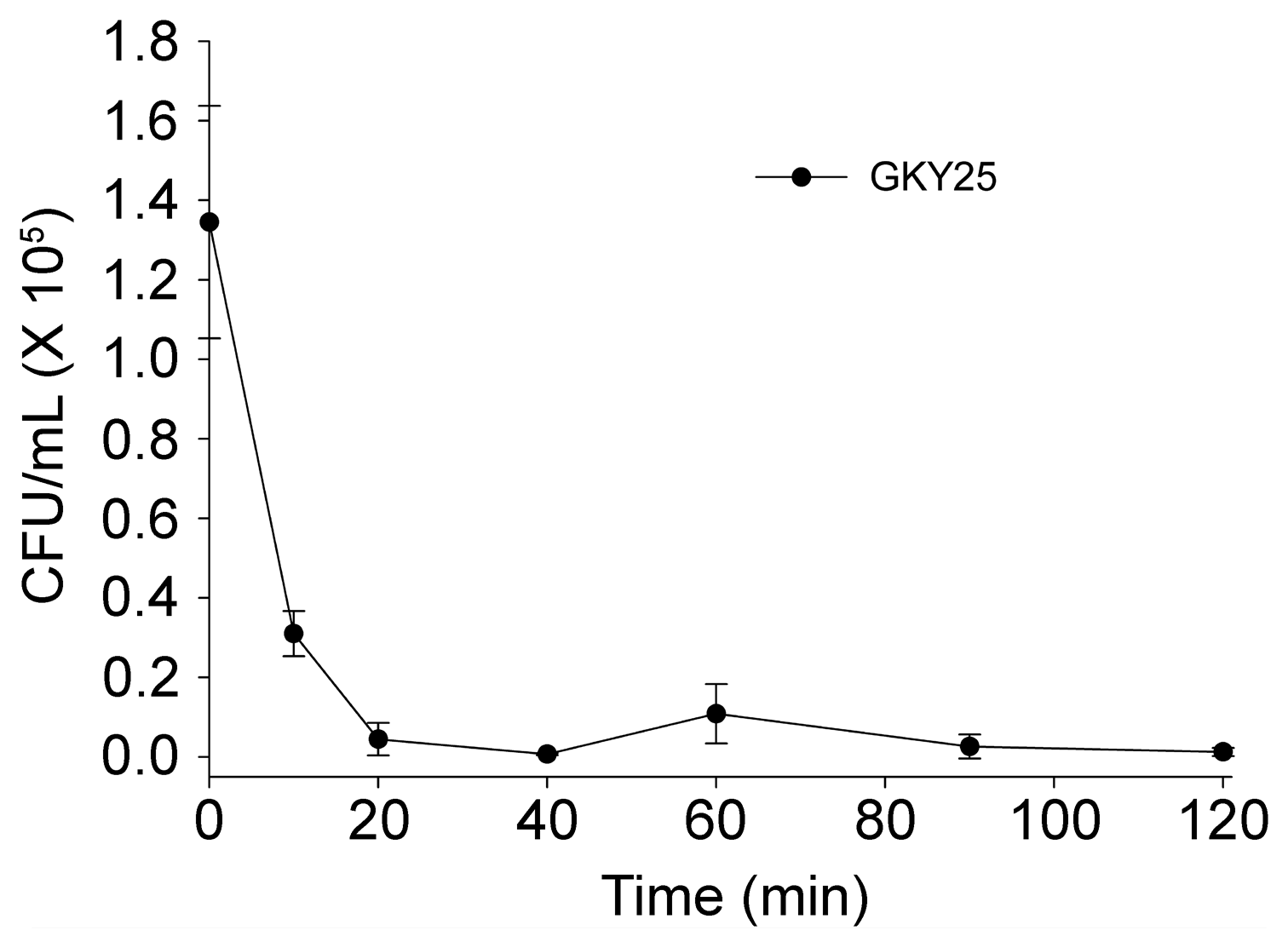

Supplement: Figure S7 — Kinetics of GKY25 action. E. coli bacteria were grown to mid-logarithmic phase in Todd-Hewitt (TH) medium. They were then washed and diluted in 10 mM Tris, pH 7.4 containing 5 mM glucose. Following this, bacteria (50 ml; 2×10^6^ cfu/ml) were incubated, at 37°C for for 5, 10, 20, 40, 60 and 120 min with GKY25 at 6 µM in presence of 10 mM Tris, 0.15 M NaCl, pH 7.4. To quantify the bactericidal activity, serial dilutions of the incubation mixtures were plated on TH agar, followed by incubation at 37°C overnight and the number of colony-forming units was determined. 100% survival was defined as total survival of bacteria in the same buffer and under the same condition in the absence of peptide. (0.22 MB TIF) [file ppat.1000857.s008.tif]

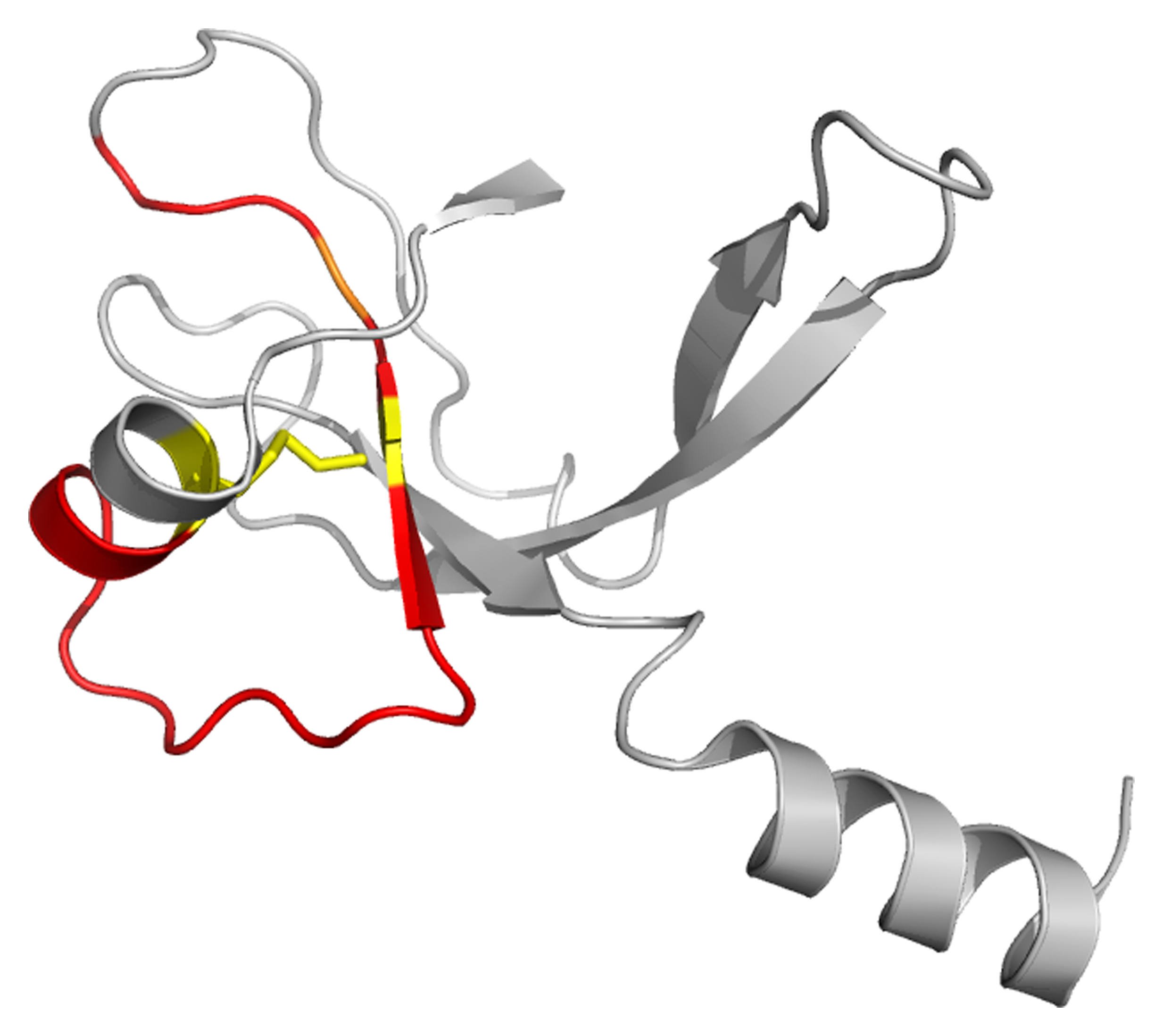

Supplement: Figure S8 — γ-core motif of TCP. Cartoon representation of the part corresponding to the C-terminal 96 amino acids of the crystal structure of thrombin (PDB code: 1C5L, amino acids 527–622). The region C536KDSTRIRITDNMFCAGYKP555 containing the proposed γ-core motif is indicated in red. Cysteines are indicated in yellow and glycines in orange. The motif corrsponds to the levomeric isoform 1 described by Yount and Yeaman (Yount, N.Y. & Yeaman, M.R. Multidimensional signatures in antimicrobial peptides. Proc Natl Acad Sci U S A 101, 7363–7368 (2004)); (NH2…[C]-[X13]-[CXG]-[X2]-P…COOH), and is quite similar to the γ-core motif found in kinocidins (Yeaman, M.R & Yount, N.Y. Unifying themes in host defence effector polypeptides. Nat Rev Microbiol. 5, 727–740 (2007)). (1.35 MB TIF) [file ppat.1000857.s009.tif]

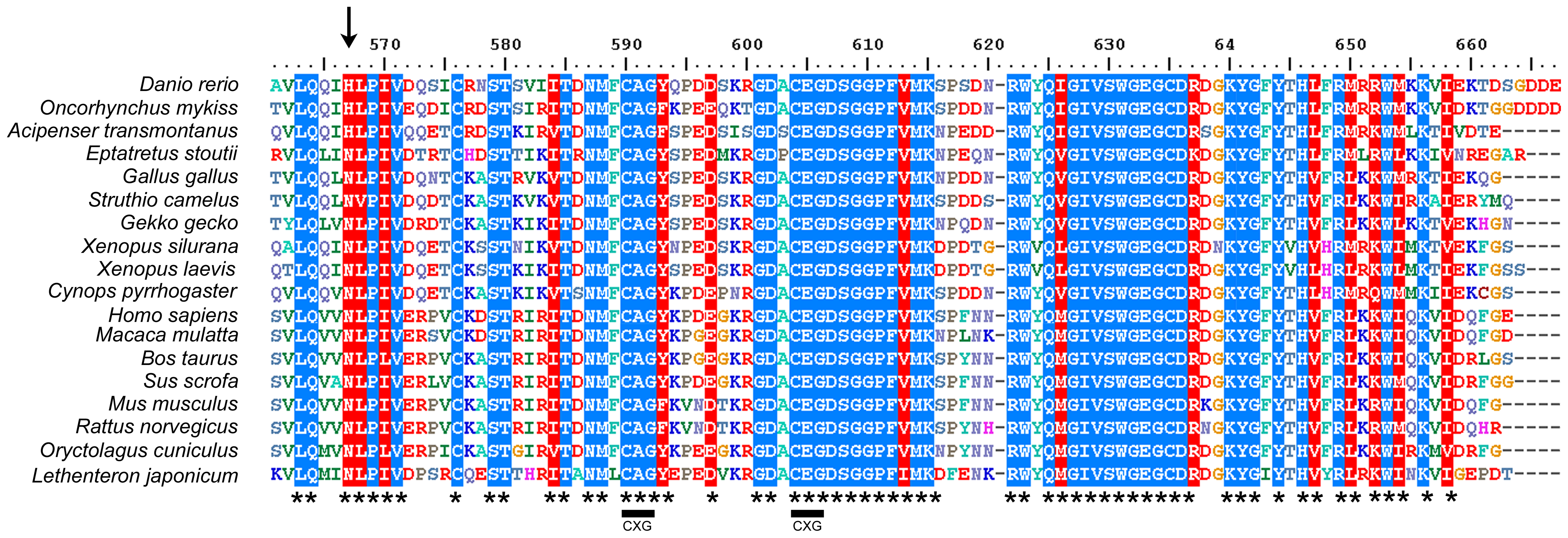

Supplement: Figure S9 — Alignment of human TCP with related thrombin sequences. The conserved cysteine residues, as well as two CXG motifs are indicated. Arrow indicates the N-terminus of the 96 amino acid peptide generated by neutrophil elastase. (7.95 MB TIF) [file ppat.1000857.s010.tif]
