# Supplementary material for: Proteolysis of Human Thrombin Generates Novel Host Defense Peptides
Source: PLoS Pathog. 2010 Apr 22;6(4):e1000857. doi: 10.1371/journal.ppat.1000857 (PMC2858699; doi:10.1371/journal.ppat.1000857)
Supplement: Table S1 — Peptide sequences of fraction 20–21. Masses were obtained by MALDI-MS analysis, and possible peptide sequences from the prothrombin sequence were deduced using the FINDPEPT tool (www.expasy.org/tools/findpept.html). (0.03 MB DOC) [file ppat.1000857.s011.doc]

**Table S1. Peptide sequences of fraction 20-21.** Masses were obtained by MALDI-MS analysis, and possible peptide sequences from the prothrombin sequence were deduced using the FINDPEPT tool ([www.expasy.org/tools/findpept.html](http://www.expasy.org/tools/findpept.html)).

| **MALDI mass** | | **DB mass** | **Δ mass (daltons)** | **Peptide** |
| --- | --- | --- | --- | --- |
| Fraction 20 | | | | |
| 975.000 | | 974.517 | -0.483 | (Y)KGRVTGWGN(L) |
| 1581.510 | | 1581.869 | 0.358 | (Y)GFYTHVFRLKKW(I) |
| 1581.510 | | 1581.974 | 0.463 | (T)HVFRLKKWIQKV(I) |
| 2243.000 | | 2243.089 | 0.088 | (F)VMKSPFNNRWYQMGIVSW(G) |
| 2502.000 | | 2502.108 | 0.107 | (K)SPFNNRWYQMGIVSWGEGCD R(D) |
| 4076.000 | | 4075.846 | -0.153 | (V)MKSPFNNRWYQMGIVSWGEG CDRDGKYGFYTHVF(R) |
| Fraction 21 | | | | |
| 975.000 | 974.517 | | -0.483 | (Y)KGRVTGWGN(L) |
| 1189.510 | 1189.579 | | 0.068 | (K)YGFYTHVFR(L) |
| 1739.690 | 1739.796 | | 0.105 | (F)NNRWYQMGIVSWGE(G) |
| 2536.090 | 2536.331 | | 0.240 | (A)SLLQAGYKGRVTGWGNLKET WTA(N) |
