# Supplementary material for: Proteolysis of Human Thrombin Generates Novel Host Defense Peptides
Source: PLoS Pathog. 2010 Apr 22;6(4):e1000857. doi: 10.1371/journal.ppat.1000857 (PMC2858699; doi:10.1371/journal.ppat.1000857)
Supplement: Table S2 — Minimal inhibitory concentrations (MIC) of GKY25, LL-37 and omiganan against various bacterial isolates. The analysis was performed as described in Wiegand et al. [39] and according to NCSLA guidelines. Additional clinical isolates were obtained from the Department of Bacteriology, Lund University Hospital. P. aeruginosa, E. coli and E. faecalis isolates were initially derived from patients with chronic ulcers, S. aureus from patients with atopic dermatitis. The S. pyogenes strain AP1 was from the WHO Collaborating Center for References and Research on Streptococci (Prague, Czech Republic). (0.04 MB DOC) [file ppat.1000857.s012.doc]

**Table S2. Minimal inhibitory concentrations (MIC) of GKY25, LL-37 and omiganan against various bacterial isolates.** The analysis was performed as described in Wiegand *et al.*  and according to NCSLA guidelines. Additional clinical isolates were obtained from the Department of Bacteriology, Lund University Hospital. *P. aeruginosa*, *E. coli* and *E. faecalis* isolates were initially derived from patients with chronic ulcers, *S. aureus* from patients with atopic dermatitis. The *S. pyogenes* strain AP1 was from the WHO Collaborating Center for References and Research on Streptococci (Prague, Czech Republic).

| Bacteria |  | MIC in (μM) | | |
| --- | --- | --- | --- | --- |
|  |  | GKY25 | LL-37 | Omiganan |
| *E. coli* | ATCC 25922 | 2.5 | 20 | 20 |
|  | Clinical isolate 37.4 | 2.5 | 5 | 20 |
|  | Clinical isolate 47.1 | 1.2 | 5 | 20 |
|  | Clinical isolate 49.1 | 10 | 10 | 10 |
| P. aeruginosa | ATCC 27853 | 160 | 10 | 160 |
|  | Clinical isolate 15159 | 20 | 20 | 20 |
|  | Clinical isolate 13.2 | 80 | 10 | 40 |
|  | Clinical isolate 27.1 | 20 | 10 | >160 |
|  | Clinical isolate 23.1 | 40 | 20 | 40 |
|  | Clinical isolate 10.5 | 20 | 10 | 40 |
|  | Clinical isolate 51.1 | 80 | 40 | 80 |
|  | Clinical isolate 62.1 | 20 | 20 | 20 |
|  | Clinical isolate 18488 | 10 | 20 | 20 |
| *S. aureus* | ATCC 29213 | 10 | 40 | 10 |
|  | FDA 486 | 10 | 10 | 20 |
|  | Clinical isolate 1088 | 10 | 160 | 20 |
|  | Clinical isolate 1090 | 10 | 160 | 80 |
|  | Clinical isolate 1086 | 80 | 20 | 10 |
|  | Clinical isolate 16065 | 2.5 | 10 | 5 |
|  | Clinical isolate 13430 | 10 | 20 | 10 |
|  | Clinical isolate 14312 | 10 | 10 | 20 |
|  | Clinical isolate 18800 | 2.5 | 5 | 2.5 |
|  | Clinical isolate 18319 | 2.5 | 10 | 20 |
| E. faecalis | Clinical isolate 2374 | 20 | >160 | 160 |
| S. pyogenes | AP1 | 2.5 | 1.2 | 5 |
