# Supplementary material for: Proteolysis of Human Thrombin Generates Novel Host Defense Peptides
Source: PLoS Pathog. 2010 Apr 22;6(4):e1000857. doi: 10.1371/journal.ppat.1000857 (PMC2858699; doi:10.1371/journal.ppat.1000857)
Supplement: Table S3 — Sequences of coagulation factor-derived peptides. (0.02 MB DOC) [file ppat.1000857.s013.doc]

**Table S3. Sequences of coagulation factor-derived peptides.**

| Protein | Designation | Sequence | net charge |
| --- | --- | --- | --- |
| Thrombin (FII) | GKY25 VFR17 | GKYGFYTHVFRLKKWIQKVIDQFGE VFRLKKWIQKVIDQFGE | +3  +2 |
| FX | GKY25(X) | GKYGIYTKVTAFLKWIDRSMKTRGL | +5 |
| FIX | GKY23 | GKYGIYTKVSRYVNWIKEKTKLT | +5 |
| FXI | ERP23 | ERPGVYTNVVEYVDWILEKTQAV | -2 |
| Kallikrein | EQP25 | EQPGVYTKVAEYMDWILEKTQSSDG | -3 |
